# CIG-P: Circular Interaction Graph for Proteomics

**DOI:** 10.1186/1471-2105-15-344

**Published:** 2014-10-31

**Authors:** Christopher K Hobbs, Michelle Leung, Herbert H Tsang, H Alexander Ebhardt

**Affiliations:** Applied Research Lab, Faculty of Natural and Applied Sciences, Trinity Western University Canada, Langley, BC Canada; Institute of Molecular Systems Biology, Department of Biology, ETH Zürich, Wolfgang-Pauli-Str. 16, 8093 Zürich, Switzerland

**Keywords:** Proteomics, Affinity purification coupled to mass spectrometry, Visualization, Integration of datasets

## Abstract

**Background:**

A typical affinity purification coupled to mass spectrometry (AP-MS) experiment includes the purification of a target protein (bait) using an antibody and subsequent mass spectrometry analysis of all proteins co-purifying with the bait (aka prey proteins). Like any other systems biology approach, AP-MS experiments generate a lot of data and visualization has been challenging, especially when integrating AP-MS experiments with orthogonal datasets.

**Results:**

We present Circular Interaction Graph for Proteomics (*CIG-P*), which generates circular diagrams for visually appealing final representation of AP-MS data. Through a Java based GUI, the user inputs experimental and reference data as file in csv format. The resulting circular representation can be manipulated live within the GUI before exporting the diagram as vector graphic in pdf format. The strength of *CIG-P* is the ability to integrate orthogonal datasets with each other, e.g. affinity purification data of kinase PRPF4B in relation to the functional components of the spliceosome. Further, various AP-MS experiments can be compared to each other.

**Conclusions:**

*CIG-P* aids to present AP-MS data to a wider audience and we envision that the tool finds other applications too, e.g. kinase – substrate relationships as a function of perturbation. *CIG-P* is available under: http://sourceforge.net/projects/cig-p/

**Electronic supplementary material:**

The online version of this article (doi:10.1186/1471-2105-15-344) contains supplementary material, which is available to authorized users.

## Background

**Figure 1:**
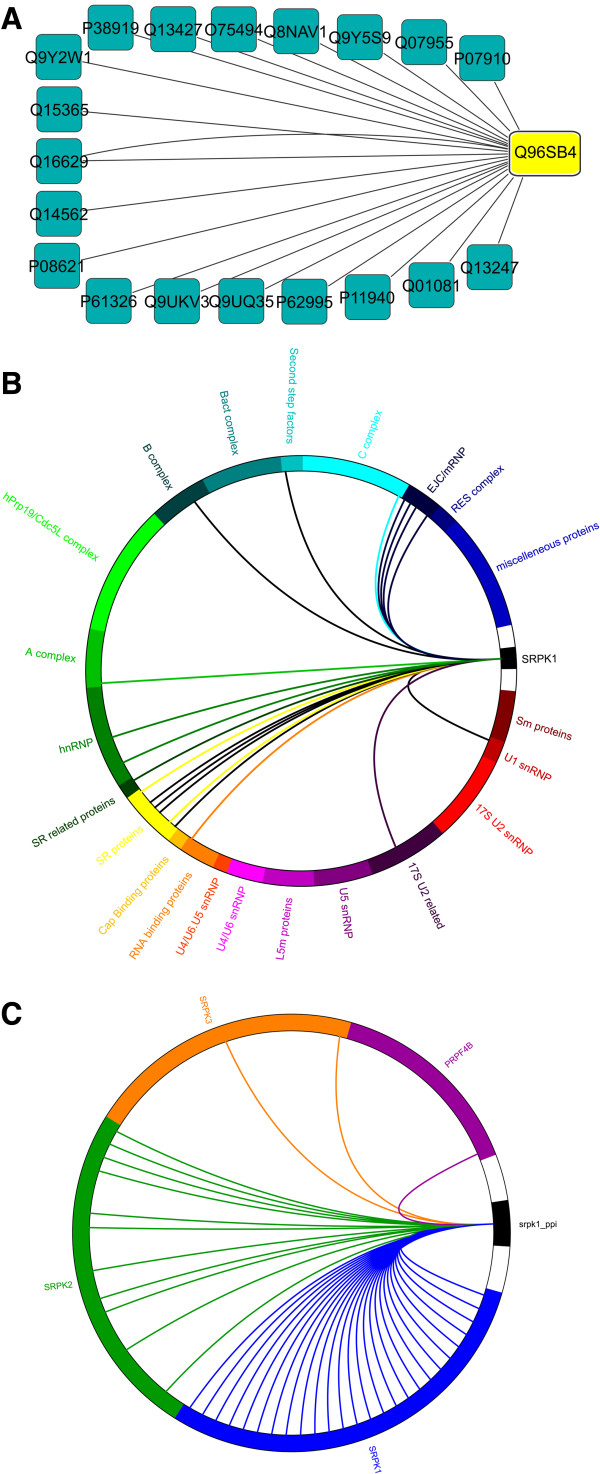
***CIG-P*** **circular diagram.** On the outer circle, all protein reference sets are placed, separated by color. The width of the colored sections is proportional to the number of proteins they encompass. As scale, the white sections have a width of 3 proteins. **A)** AP-MS data displayed in circular layout in cytoscape. **B)** The AP-MS dataset is projected onto a functionally annotated protein dataset. The interactors of SRPK1 were drawn in colored arcs while the kinase substrate data was drawn in black arcs. As seen, the interactors and kinase substrates of SRPK1 are specific to sub-complexes of the spliceosome (see main text for discussion). **C)** Lenticular function *CIG-P* diagram (reappearance filtering ON) comparing the interactors of four kinases to interactors of SRPK1. From the black section of SRPK1 (bait protein), colored arcs are drawn representing the high quality protein-protein interactions found in each AP-MS experiment. As seen from the diagram, interactors of SRPK1 and SRPK2 are similar, while SRPK3 and PRPF4B show distinct differences in their interactome.

Proteomics using high mass accuracy mass spectrometers aims to characterize the proteome of a cell over space and time [1]. A critical sub-discipline of proteomics is affinity purification coupled to mass spectrometry (AP-MS) [2]. The goal of an AP-MS experiment is to qualitatively and quantitatively characterize protein complexes by either affinity-tagging a protein of interest (bait) or using a bait-specific antibody for affinity purification. Following the enrichment of bait protein, bait-associated proteins will co-purify (termed: prey or interactors) and are identified by subsequent standard bottom-up proteomics approach using high mass accuracy mass spectrometry [3]. The digitized record of an AP-MS experiment is annotated using in silico search engines mapping MS/MS spectra to peptides in the case of shotgun-mass spectrometry [4] or mapping ion chromatograms to spectral libraries in the case of SWATH-MS [5]. Following the in silico annotation is the critical contaminant filtering to avoid CRAPome annotations [6]. Frequently used filtering algorithms include SAINT [7], Abacus [8] or a creative relaxed local network scoring scheme [9]. A software package which aids the AP-MS researcher is *Cytoscape* [10], which is used to integrate, interrogate and filter AP-MS data as well as provide links to functionally annotated databases. The final product of this extensive AP-MS workflow is a high quality, high confidence protein network with edges and nodes frequently presented using the visual output of *Cytoscape* (see Figure 1A). Although familiar to the AP-MS scientist, these edges and nodes containing diagrams are not always easy to grasp at first glance by a wider audience. A further limitation of current AP-MS visualization is the abstract integration of orthogonal datasets for relative interpretation of the newly created dataset. For conveying genomic information, *Cirocs* was introduced by Krzywinski *et al.* [11]. Although *Circos* is very versatile, it also has a very complex input data structure, which includes a very steep learning curve prior to using the program. To simplify the generation of aesthetic comparative proteomics and to overcome the graphical limitation of *Cytoscape*, we developed Circular Interaction Graph for Proteomics (*CIG-P*). We believe the circular representation is a very intuitive way of conveying the information content to a wider audience. Through abstracting the data, we further hypothesize that scientist will be able to easier interpret their high quality, high confidence protein networks data.

## Implementation

For generating circular diagrams, we developed an intuitive Java based graphical user interface termed *CIG-P* which uses three input files: an experiment file containing the actual AP-MS experiment, a reference file containing a dataset to which the experiment is compared to and lastly a RBG color scheme (see Additional files 1, 2, 3, 4, 5 for examples). In detail, the experiment file contains three comma delimited columns. The first column contains a unique string identifying the bait protein, the second column contains a coding if the drawn arc should be colored (‘ppi’) or black (‘ivtk’), while the third column contains a unique string identifying the prey proteins. The reference file is also comprised of three comma delimited columns whereby columns 1 and 3 are of descriptive nature, while column 2 contains a unique protein identifier string and will be mapped to column 3 of the experiment file. In our case, we use unique uniprot IDs to be consistent within the dataset. The reference file also defines *protein sets* which are identified similar to the FASTA format initiating the *protein set* with an ‘>’. Lastly, the color scheme is coded in a highly customizable comma delimited RBG format. The number of colors is dependent on the dataset ideally one color per *protein set* should be used. In case the *protein sets* defined in the reference file outnumber the colors listed in the color scheme, latter will be cycled until all *protein sets* are colored. Knowing the RBG color code of each *protein set* will also make subsequent integration of *CIG-P* diagrams into presentations easier, as add-on fonts or drawings can take advantage of matching colors. More details on the organization of these files can be found in the (Additional file 2) User_Manual.

## Results and Discussion

The output of *CIG-P* is a circular diagram. On the outer circle of the diagram, both the reference *protein sets* and the bait protein are placed, latter is flanked by white spaces serving as separator and scale as each white space is proportional to three proteins. The interactions defined in the experiment file are drawn as arcs in the center of the circle (see Figure 1B and C). We believe this layout is quite intuitive and conveys the nature of an AP-MS experiment, whereby all interactions represented by arcs originate from the bait protein. The initial default settings of circle size and arc thickness can be adjusted using the controls in the top left of the *CIG-P*’s graphical user interphase. Also, new experiment, reference or color schemes can be loaded live into the newly drawn circular diagram. Following below, we present two distinct applications of *CIG-P*: first the quick visualization of various AP-MS experiments to each other, while the second application focuses on the visual integration of orthogonal proteomics datasets.

For the lenticular function *CIG-P* diagram, the *protein sets* in the reference input file are defined as high confidence prey proteins of individual baits, comparing multiple baits with each other. Alternatively, each *protein set* can be defined as set of high-confidence prey proteins per condition of the same bait where the cellular system underwent a perturbation. For example, the primary *protein set* is defined as the prey proteins of a particular bait when the cellular system is treated with the carrier, while all subsequent *protein sets* are the prey proteins upon stimulating the cellular system with a particular chemical compound. The resulting series of *CIG-P* circular diagrams will rapidly visualize the changes in the interactome of the bait as a function of perturbation.

*CIG-P* is also equipped with a reappearance mapping function. If turned *OFF*, only the first instance of a match is mapped and displayed as arc. The reappearance function *OFF* can be useful in the above mentioned scenario of a perturbed cellular system whereby the primary *protein set* contains all prey proteins of the control, while all other *protein sets* contain prey proteins under perturbation. This set up allows for visualizing which prey proteins are new compared to the control AP-MS experiment (see Additional file 1: Figures S7-S10). On the other hand, with the reappearance function *ON* all interactions are redundantly drawn, which is important if multiple reference *protein sets* contain the same protein, e.g. if a certain protein belongs to multiple functionally annotated protein complexes (see **Application of*CIG-P*** below).

Great insight into individual AP-MS experiments can be gained by projecting the newly generated data onto orthogonal proteomics datasets. Orthogonal datasets could include native protein complex fractionation techniques [12] or functional fractionations and annotations of super-complexes, such as the ribosome, proteasome or spliceosome [13]. Using this type of higher order annotation, the individual AP-MS experiment is immediately placed into a wider context for rapid interpretation of the data at hand.

To demonstrate the functionality of *CIG-P*, we visualize data of a published dataset [14] and draw conclusions from our circular diagrams which were not mentioned in the original publication, supporting our initial motivation that abstract visualization can guide scientists to establish new working hypothesis.

The original dataset [14] encompasses the interactome of the CMGC clade of kinases. Four members of this CMGC clade show many interactions with splicing related proteins. Hence, we will focus on these four kinases: SRPK1 (Uniprot ID: Q96SB4), SRPK2 (P78362), SRPK3 (Q9UPE1) and PRPF4B (Q13523). Although, all kinases mentioned are associated with the splicosome, latter is an extremely dynamic ribonucleic complex catalyzing the excision of exons from a primary messenger RNA. To visualize that some of these kinases with overlapping prey proteins, we used the lenticular function of *CIG-P* and defined as *protein sets* (reference file) the preys associated with each kinase. When loading the experiment file of SRPK1 in the non-redundant mapping mode, all 26 interactors are visualized (Additional file 1: Figure S2). To immediately visualize the overlap of the SRPK1 interactome with the prey proteins of the other kinases, the reappearance function of *CIG-P* was turned on (Additional file 1: Figure S7). From the redundant circular diagram it is apparent that SRPK1 and SRPK2 share a lot of prey proteins, while SRPK3 and PRPF4B have a distinct interactome.

To illustrate the distinct nature of PRPF4B its experiment file is loaded into *CIG-P* from the graphical user interface. It is apparent from the circular representation (see Additional file 1: Figures S5 and S10) that PRPF4B has a distinct interactome presumably acting on a subset of spliceosomal proteins within the splicing cascade.

To follow up on commonalities and differences of these four kinases with spliceosomal prey proteins, we set as reference list a *protein set* derived from extensive functional fractionation of some 300 spliceosomal proteins [13]. The projection of AP-MS data onto an orthogonal proteomics dataset allows scientist to place the AP-MS data into context (see Figure 1B). As already established in the lenticular function *CIG-P*, SRPK1 and SRPK2 share largely an overlapping network of interactors throughout the splicing cycle, except a complete lack of interactors from the tri-snRNP (U5.U4/U6) (see Additional file 1: Figures S15 and S16). On the contrary, PRPF4B almost exclusively interacts with tri-snRNP associated proteins (see Additional file 1: Figure S18). From the lenticular function *CIG-P* analysis it was expected that the interactors were quite dissimilar, however, projecting the AP-MS dataset onto an orthogonal functionally fractionated proteomics dataset allows for a rapid functional annotation and visualization of these differences.

Besides rapid comparison of different kinase interactors, integration of orthogonal proteomics datasets, *CIG-P* can also serve to create new working hypothesis: for SRPK1 and SRPK2 not only the prey proteins were determined, but also the in vitro kinase substrates [14]. Hence, we took advantage of *CIG-P*’s function to either draw colored or black arcs (as defined in the experiment file). We define colored arcs as protein-protein interactions and black arcs as protein kinase substrates (see Figure 1B). In the case of SRPK1 we postulate that the kinase binds to 17S U2 related proteins and phosphorylates a U1 snRNP protein, presumably promoting a dynamic transition at the onset of the splicing process.

## Conclusions

*CIG-P* assists the molecular systems biologist with AP-MS data to rapidly interrogate the high quality high confidence AP-MS prey protein dataset. Various AP-MS experiments can be compared to each other, while the integration of AP-MS data with orthogonal proteomics datasets allows the generation of statements with biological context and intuitive images suitable for a wide audience.

## Availability and requirements

**Project name:***CIG-P* Circular Interaction Graph for Proteomics

**Project home page:**http://sourceforge.net/projects/cig-p/

**Operating system:** Platform independent

**Programming language:** Processing

**Other requirements:** Java version Java7v45

**License:** GNU GPL

**Any restrictions to use by non-academics:** N/A

Source code is available upon request from herbert.tsang@twu.ca.

## Funding

The development of CIG-P is supported by the faculty start-up grant of Trinity Western University. H.A.E. is a Marie Curie International Incoming Fellow (FP7).

## Electronic supplementary material

Additional file 1: **Supporting Figures S1-S18.** (PDF 911 KB)

Additional file 2: **User Manual for** ***CIG-P*** **.** (PDF 4 MB)

Additional file 3: **Colour_rb.csv (colors).** (CSV 231 bytes)

Additional file 4: **Complex_resolved.csv (background).** (CSV 4 KB)

Additional file 5: **SRPK1.csv (experiment).** (CSV 1 KB)

## Abbreviations

CIG-P: Circular interaction graph for proteomics
AP-MS: Affinity purification coupled to mass spectrometry
RGB: Red-green-blue color code
SRPK1-3: Serine/Arginine-Rich Splicing Factor Kinase 1-3
PRPF4B: Pre-MRNA Processing Factor 4.
