# Supplementary material for: CIG-P: Circular Interaction Graph for Proteomics
Source: BMC Bioinformatics. 2014 Oct 31;15(1):344. doi: 10.1186/1471-2105-15-344 (PMC4286935; doi:10.1186/1471-2105-15-344)
Supplement: Supplementary file 1 — Additional file 1: Supporting Figures S1-S18. (PDF 911 KB) [file 12859_2014_6683_MOESM1_ESM.pdf]

CIG-P Documentation

CIG-P visualizes data in a circular layout, which makes it easy for users to understand the interactions between specific proteins. It is developed in Processing, which can be easily compiled into executable files compatible with GNU/Linux, Macintosh, and Windows operating systems or deployed as a web applet. *CIG-P* requires three data files in csv format: the experiment file, the reference file, and the color scheme file. The simplified three file csv format is intuitive for any user and caters the proteomics researcher. The experiment file contains the experiment protein to be used at the base of the comparison. The reference file contains all of the proteins that the experiment protein is being compared to. The color scheme file is a list of RBG colors allowing you to set a custom color scheme for your data. In order to differentiate between each family of proteins, a " > " is required in front of the protein family name in the reference file. *CIG-P* will use the data from the files and draw the interactions and label each of the protein family arcs. The size of a protein family is also represented through the size of its respective protein family arc. The proportional protein family sizing along with the color coding allows the user to more easily visualize the proteins which interact and how they compare. The *CIG-P* interface allows users to rotate the image, making it easier to focus on a certain type of interaction, as well as allowing them to change data files on the fly. When the user is satisfied with the visualization the image can be exported into a scalable vector image saved in PDF form.

- Requirements: Java version Java7v45, Mac, Linux, Win compiled
- Download program from: http://sourceforge.net/projects/cig-p/
- unzip executable
- start program by double clicking **CIGp.bat**

Greeted by welcome screen:

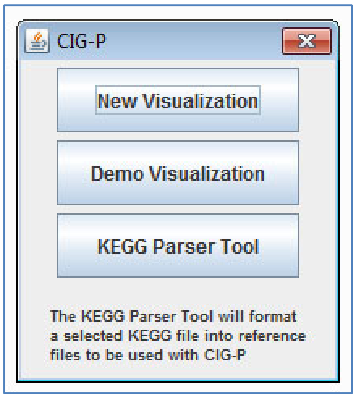

The **KEGG Parsing Tool** uses the KEGG2 Pathway Module file (e.g. ko00002.keg).

The **Demo Visualization** will create an instant circular interaction of the data used in this publication (**Figure 1B**).

For a **New Visualization**, click the appropriate button.

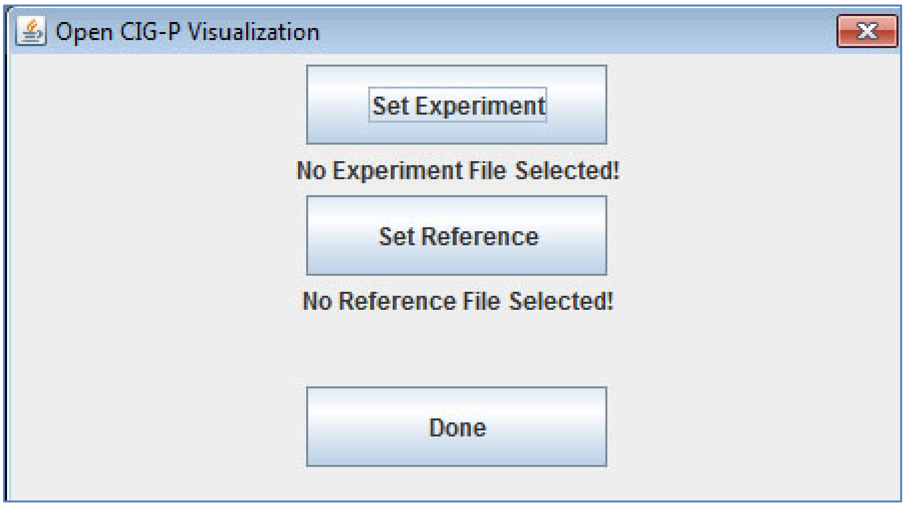

Then, to **Set Experiment**, click the appropriate button.

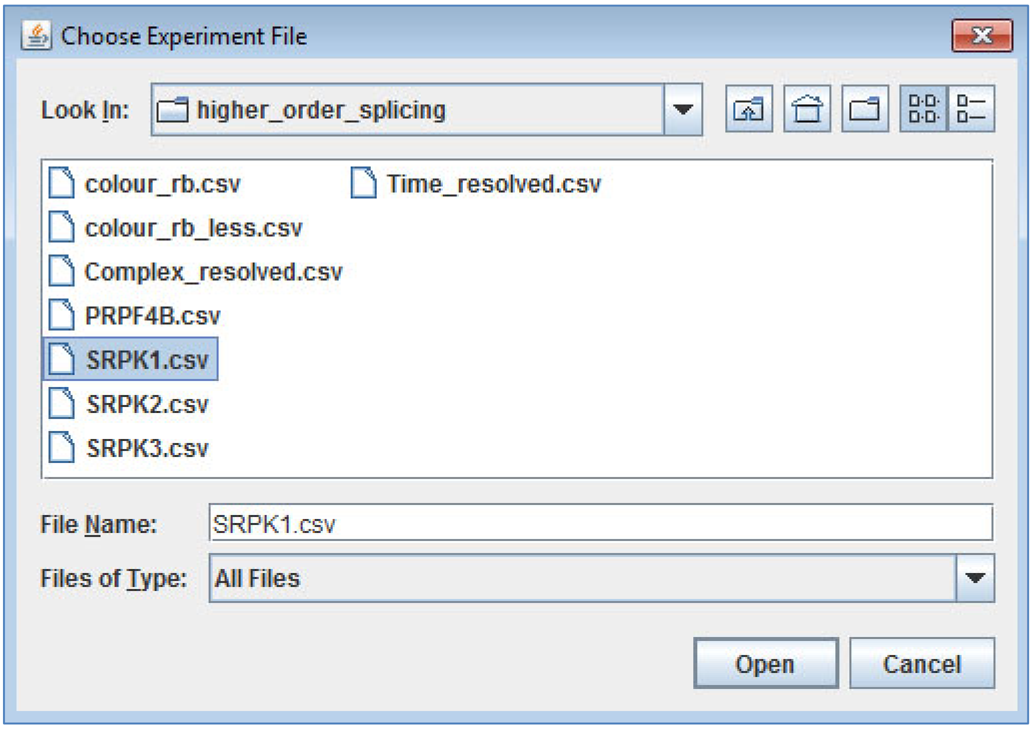

Provide these experiment files in one directory. The format for the Experiment file (e.g. bait file) is in csv format:

```
Q96SB4,ivtk,Q9HCS5
Q96SB4,ivtk,Q9NQ29
Q96SB4,ivtk,Q9NWB6
Q96SB4,ivtk,Q9P270
Q96SB4,ivtk,Q9Y383
Q96SB4,ppi,P07910
Q96SB4,ppi,P11940
Q96SB4,ppi,P18583
Q96SB4,ppi,P38919
Q96SB4,ppi,P42857
```

ppi: will generate colored lines from the bait to the outer arc
ivtk: will generate black lines from the bait to the outer arc

These csv files can also be generated in a spread sheet and saved as csv file.

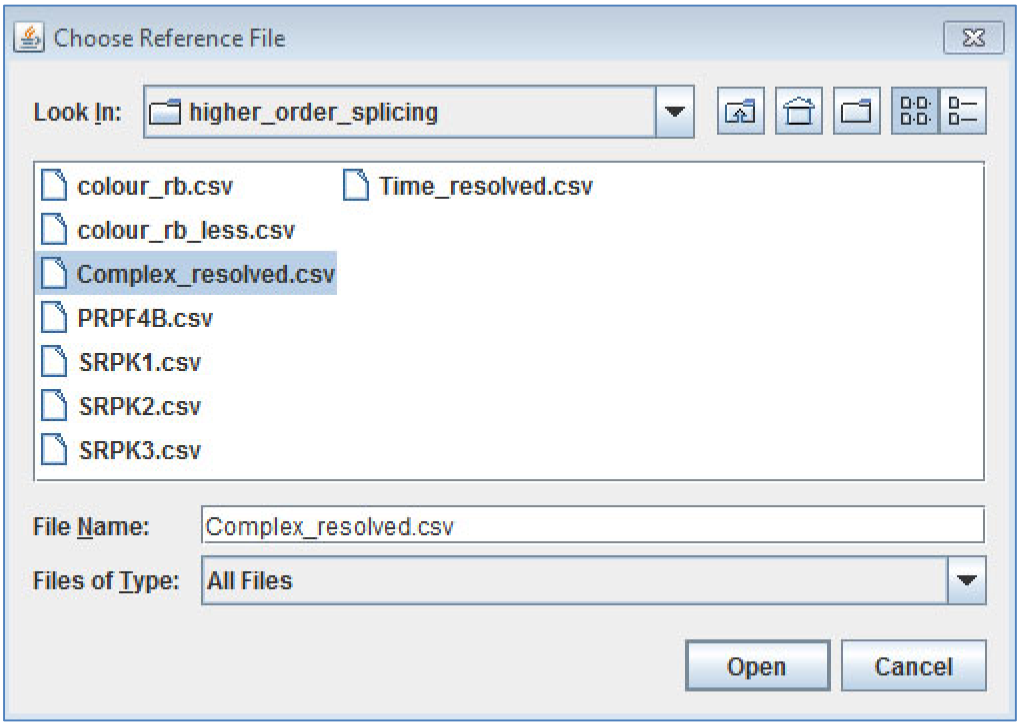

The outer arc is subdivided into various sections, each of which represents a certain *protein sets*, e.g. ribonucleic complex.

```
>Sm proteins,,
B*,P14678,SNRPB
D1,P62314,SNRPD1
D2,P62316,SNRPD2
D3**,P62318,SNRPD3
E,P62304,SNRPE
F,P62306,SNRPF
G,P62308,SNRPG
,,
>U1 snRNP,,
U1-70K,P08621,SNRNP70
U1-A,P09012,SNRPA
U1-C,P09234,SNRPC
,,
>17S U2 snRNP,,
U2A',P09661,SNRPA1
U2B,P08579,SNRPB2
SF3a120,Q15459,SF3A1
SF3a66,Q15428,SF3A2
SF3a60,Q12874,SF3A3
SF3b155,O75533,SF3B1
SF3b145,Q13435,SF3B2
SF3b130,Q15393,SF3B3
SF3b49,Q15427,SF3B4
SF3b14a/p14,Q9Y3B4,SF3B14
SF3b14b,Q7RTV0,PHF5A
SF3b10,Q9BWJ5,SF3B5
```

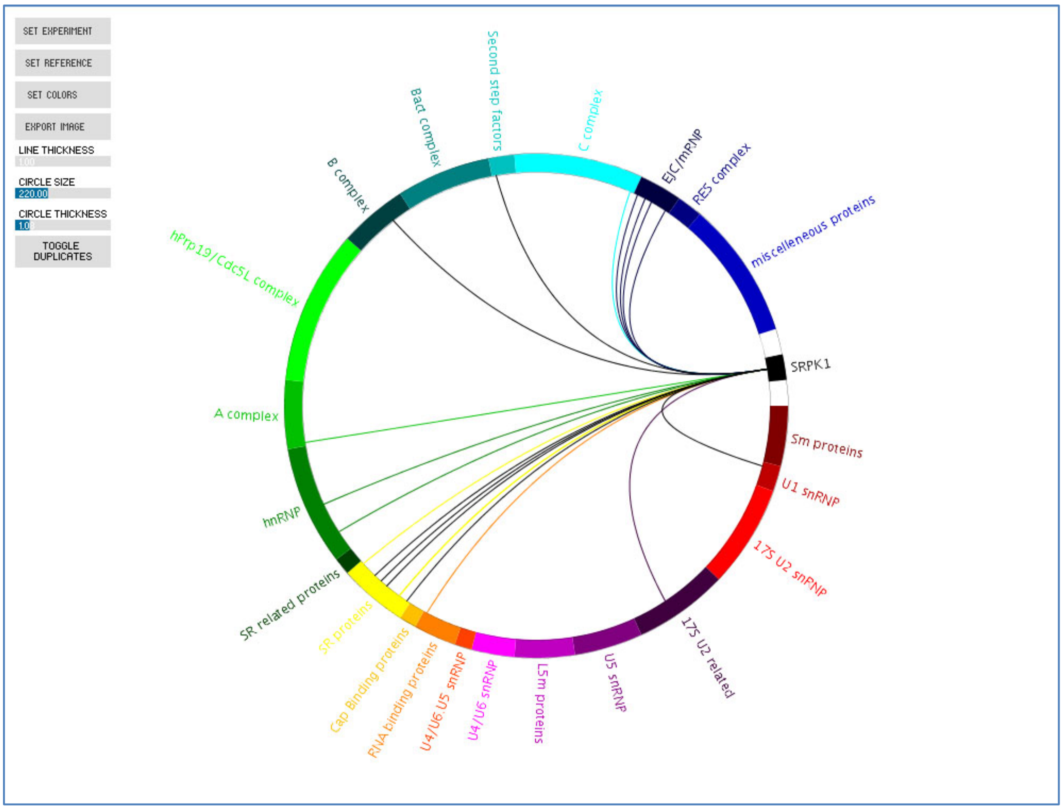

Voila: rotating of the circle is done using the mouse – moving the mouse horizontally will rotate the position of the bait.

The colour background can be changed also from within the CIG-P graphical interface (top left corner).

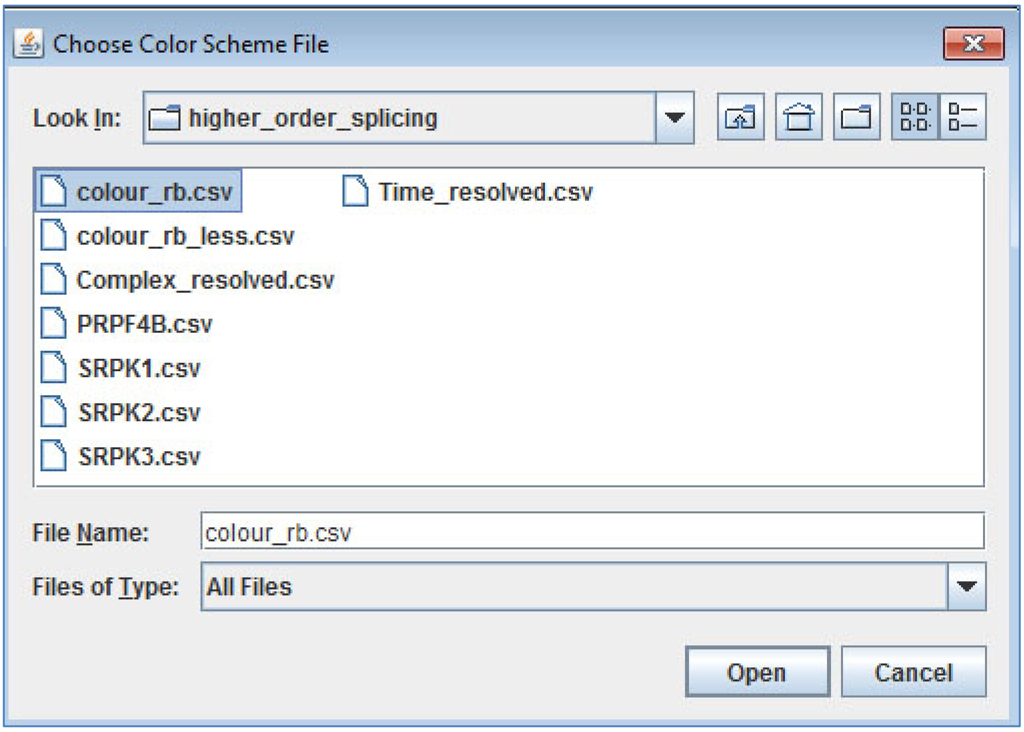

```
128,0,0
192,0,0
255,0,0
64,0,64
128,0,128
192,0,192
255,0,255
255,64,0
255,128,0
255,192,0
255,255,0
0,64,0
0,128,0
```

One the left top are all the controls, e.g. to increase the line thickness.

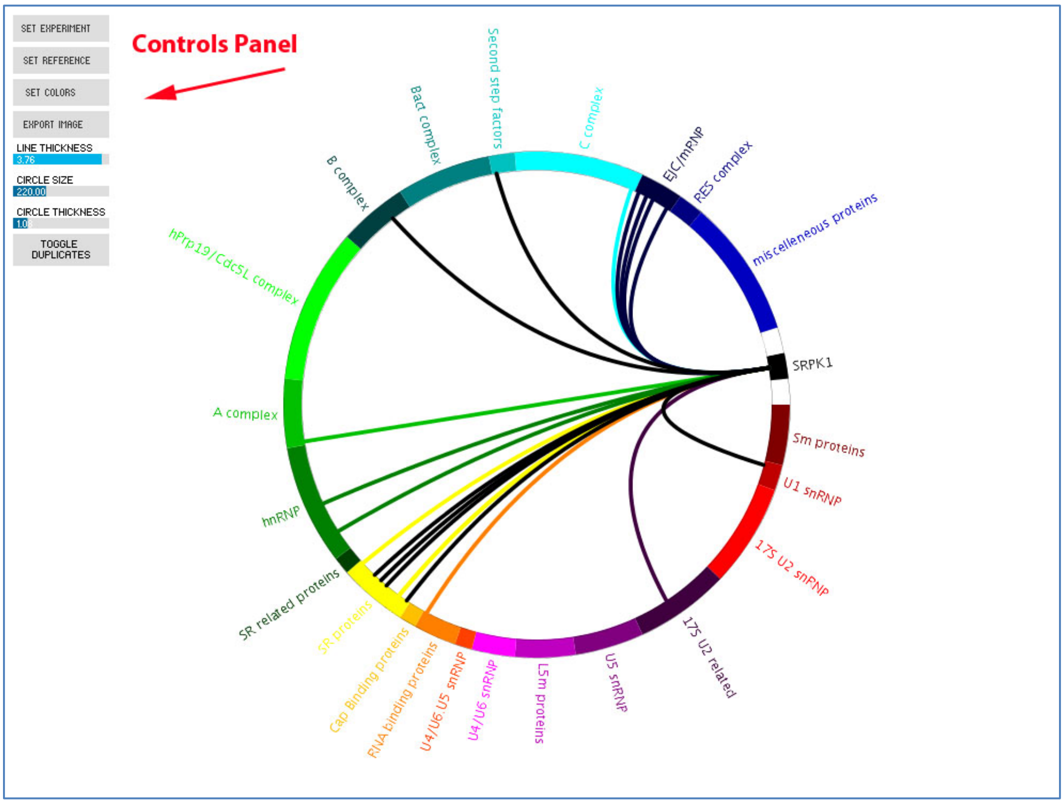

Another control function is the Reappearance OFF/ON button: when highlighted in blue, the Reappearance function is ON, while when the button is grey, the function is OFF.

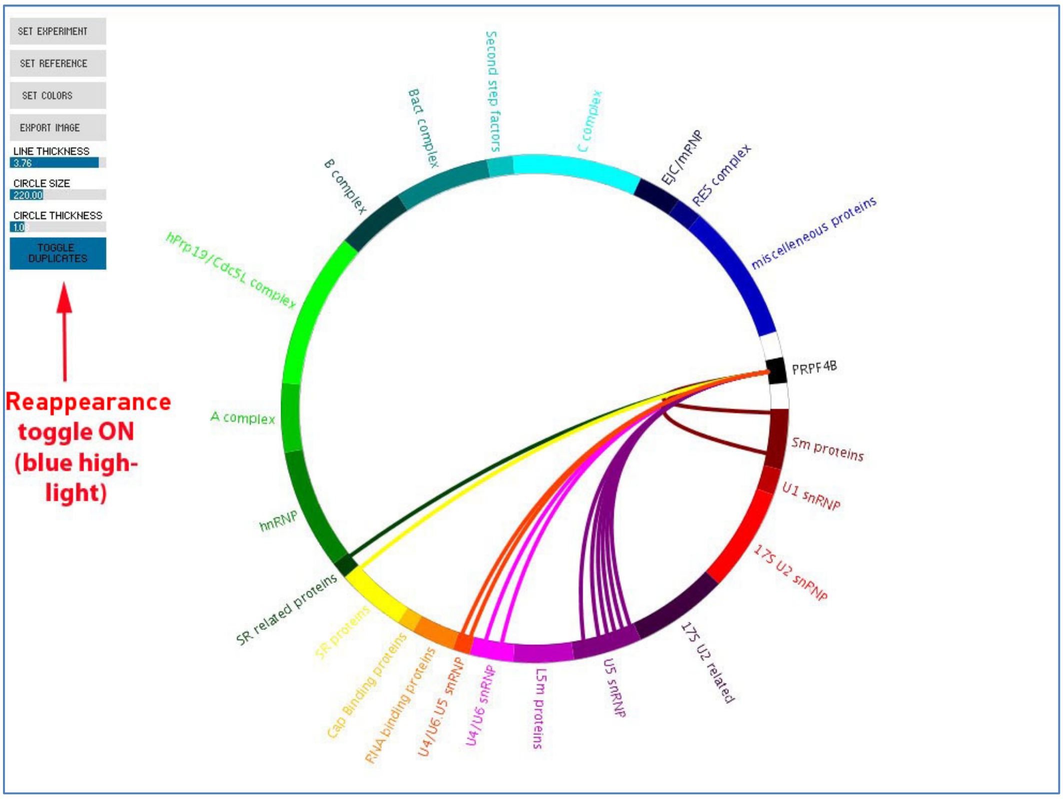

To change the experiment, e.g. bait protein data, press the SET EXPERIMENT button and select the csv file containing the different data.

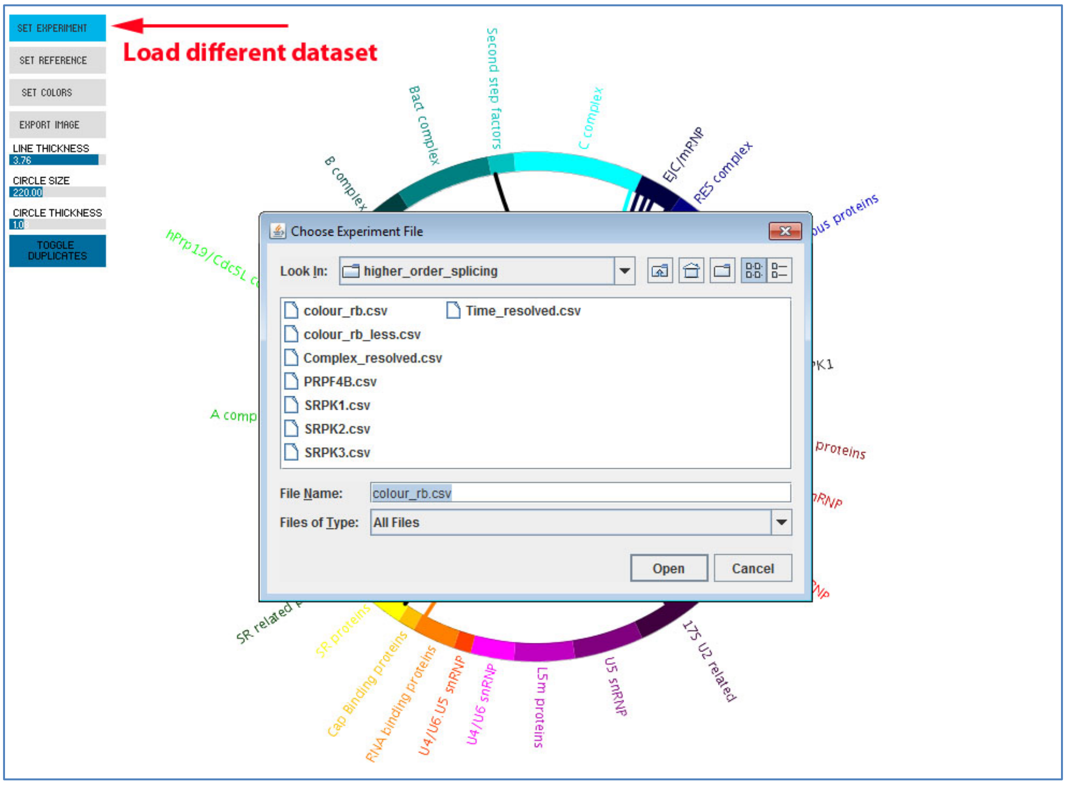

Images can be exported using the EXPORT IMAGE button, which will generate a scalable pdf file in the working directory of CIGp.bat.
