# Supplementary material for: CIG-P: Circular Interaction Graph for Proteomics
Source: BMC Bioinformatics. 2014 Oct 31;15(1):344. doi: 10.1186/1471-2105-15-344 (PMC4286935; doi:10.1186/1471-2105-15-344)
Supplement: Supplementary file 2 — Additional file 2: User Manual for CIG-P . (PDF 4 MB) [file 12859_2014_6683_MOESM2_ESM.pdf]

CIG-P circular diagram Supplementary Figure 1.

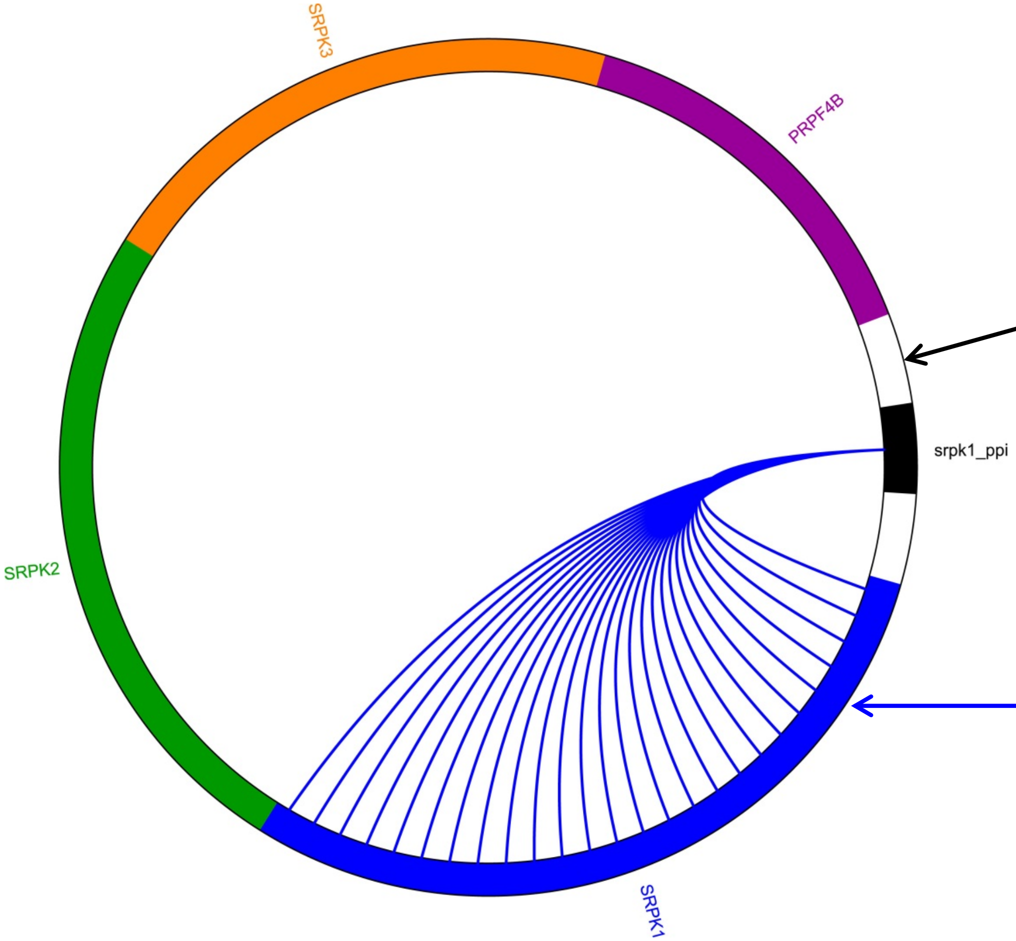

Width of white bar equals 3 proteins.

Bait protein

On arc, all 26 interacters found by affinity purification of SRPK1

Hobbs et al. unpublished.

Venn diagram vs. lenticular CIG-P Supplement F2.

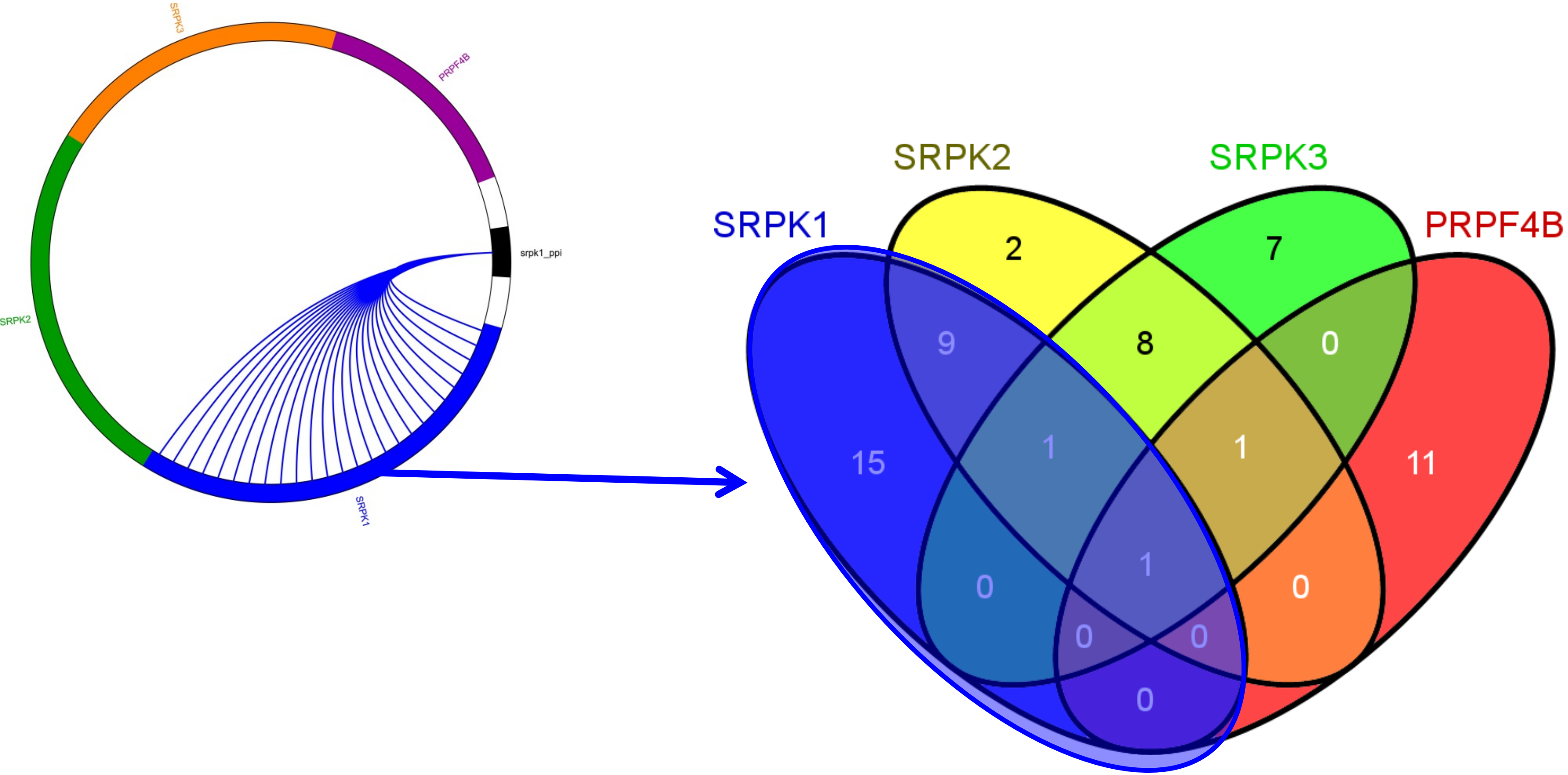

Hobbs et al. unpublished.

Venn diagram vs. lenticular `CIG-P` Supplement F3.

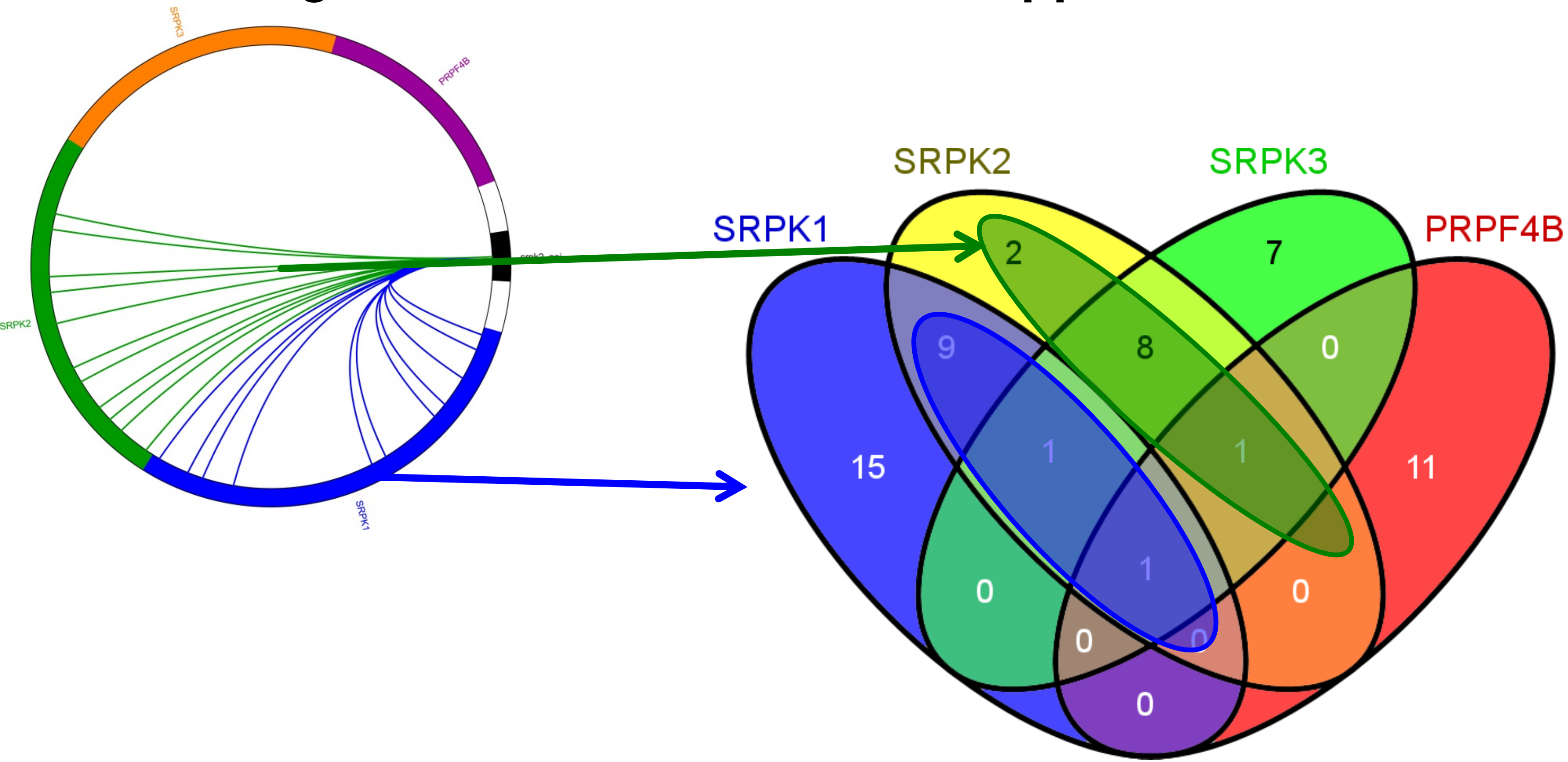

Hobbs et al. unpublished.

Venn diagram vs. lenticular `CIG-P` Supplement F4.

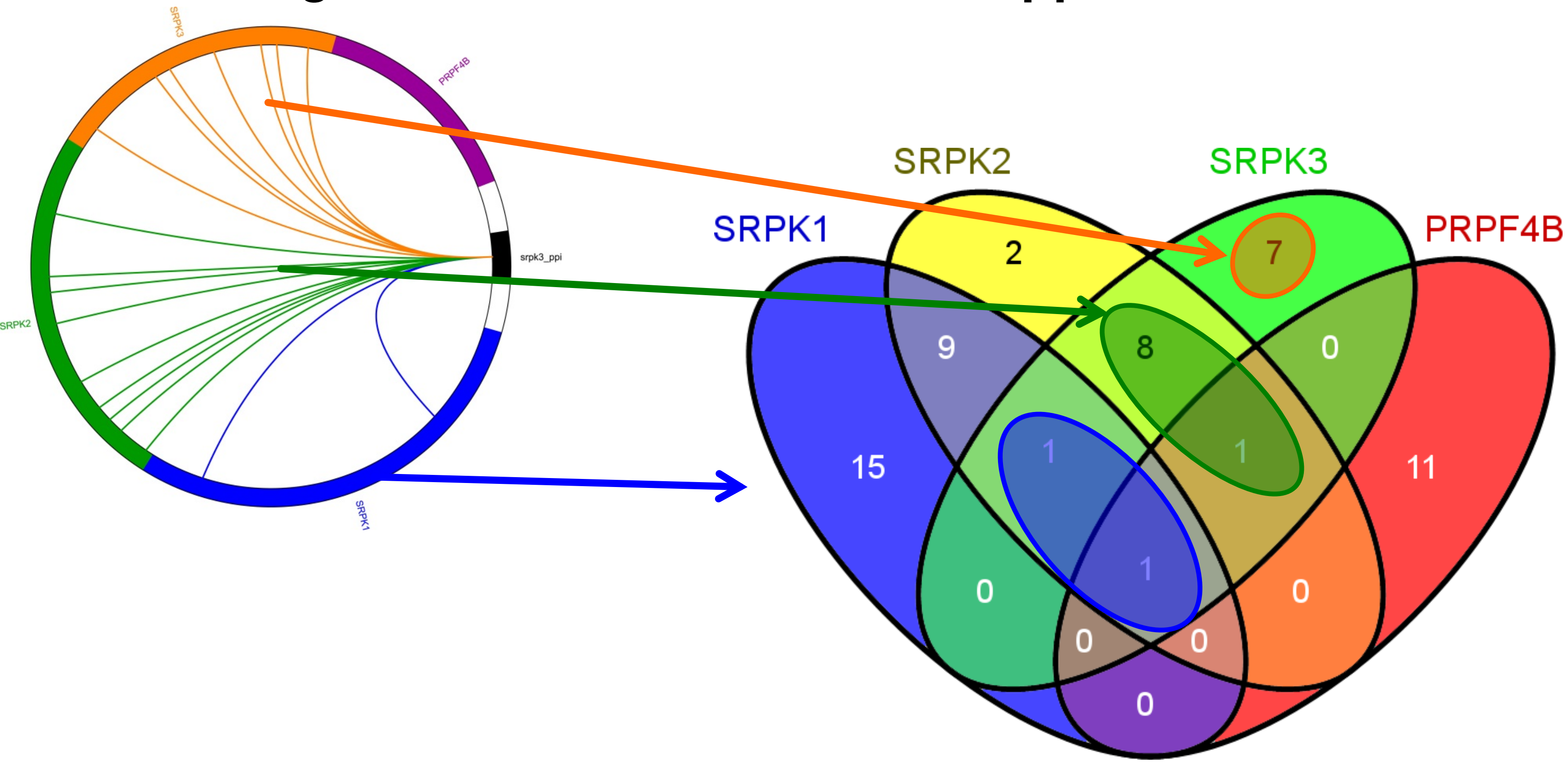

Hobbs et al. unpublished.

Venn diagram vs. lenticular CIG-P Supplement F5.

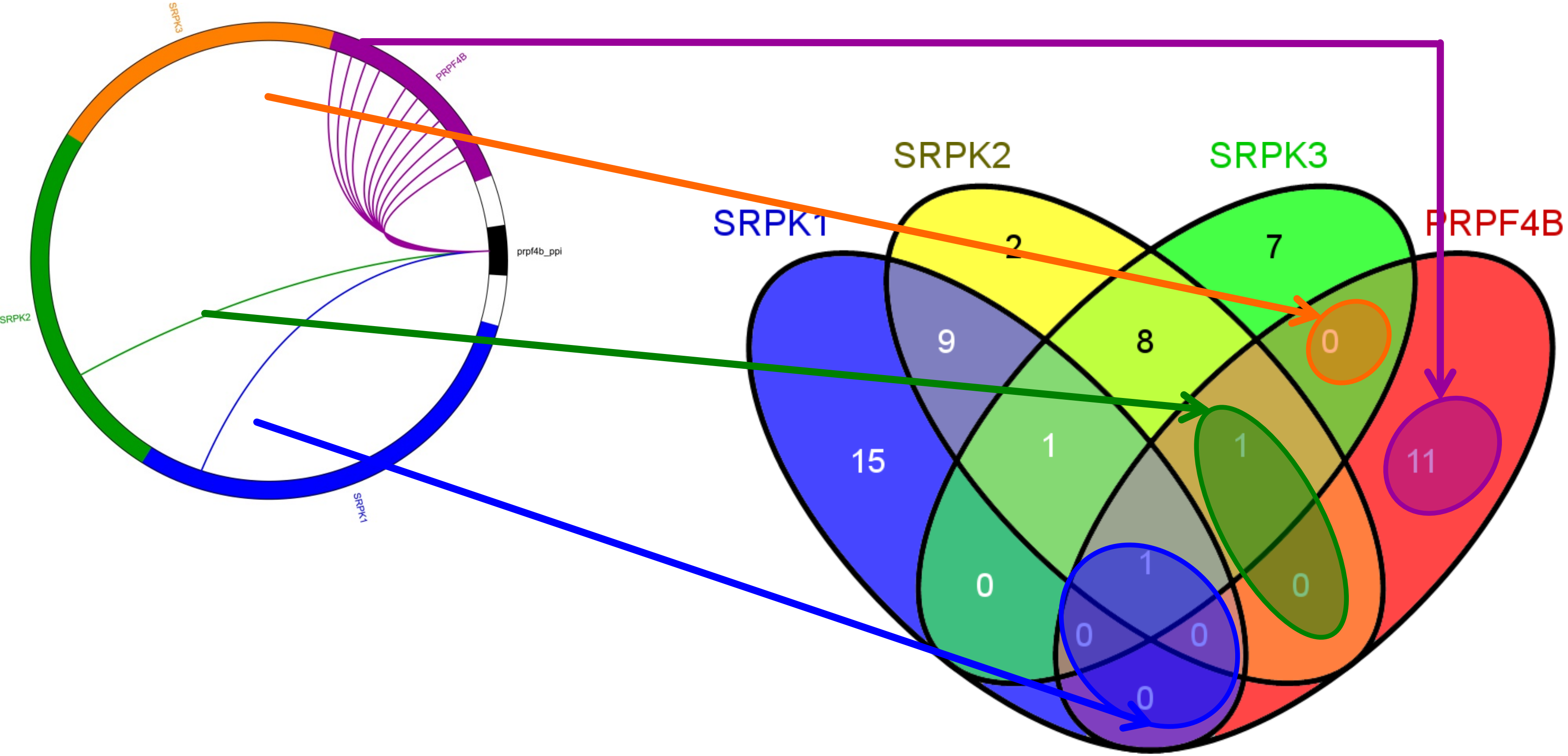

Hobbs et al. unpublished.

F6

Kinase interactors vs. kinase interactors: Reappearance filter OFF/ON.

Hobbs et al. unpublished.

Kinase interactors vs. kinase interactors: Reappearance filter OFF/ON.

F7

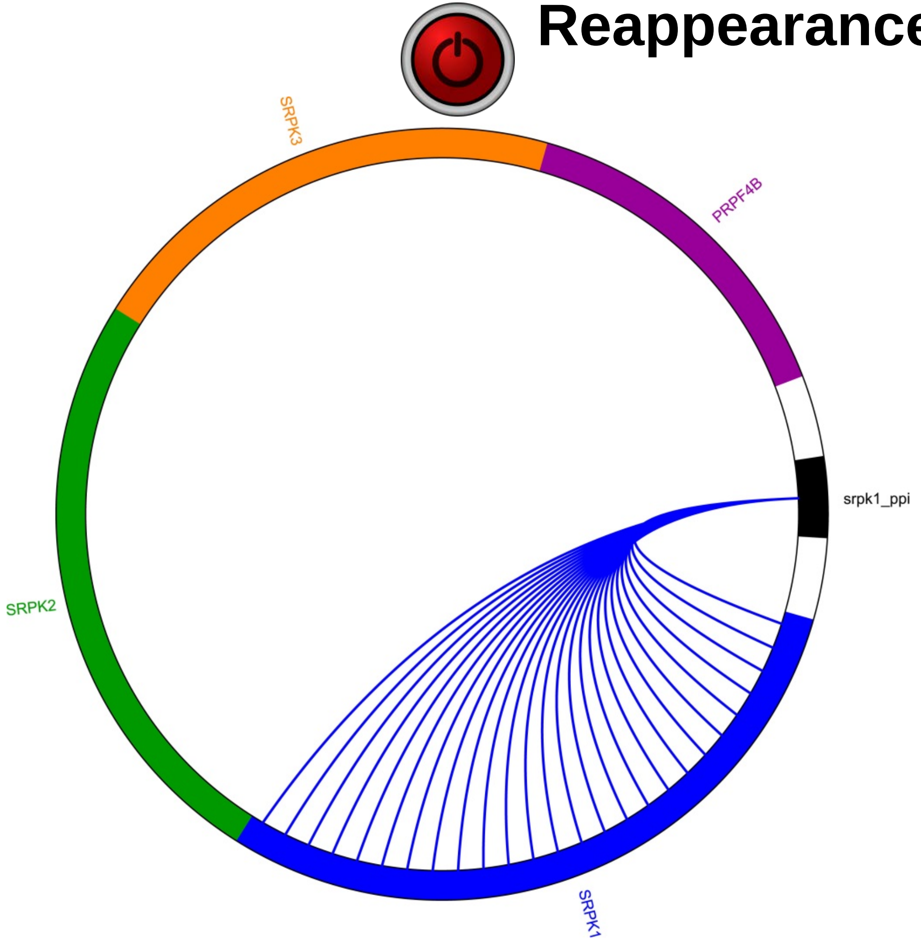

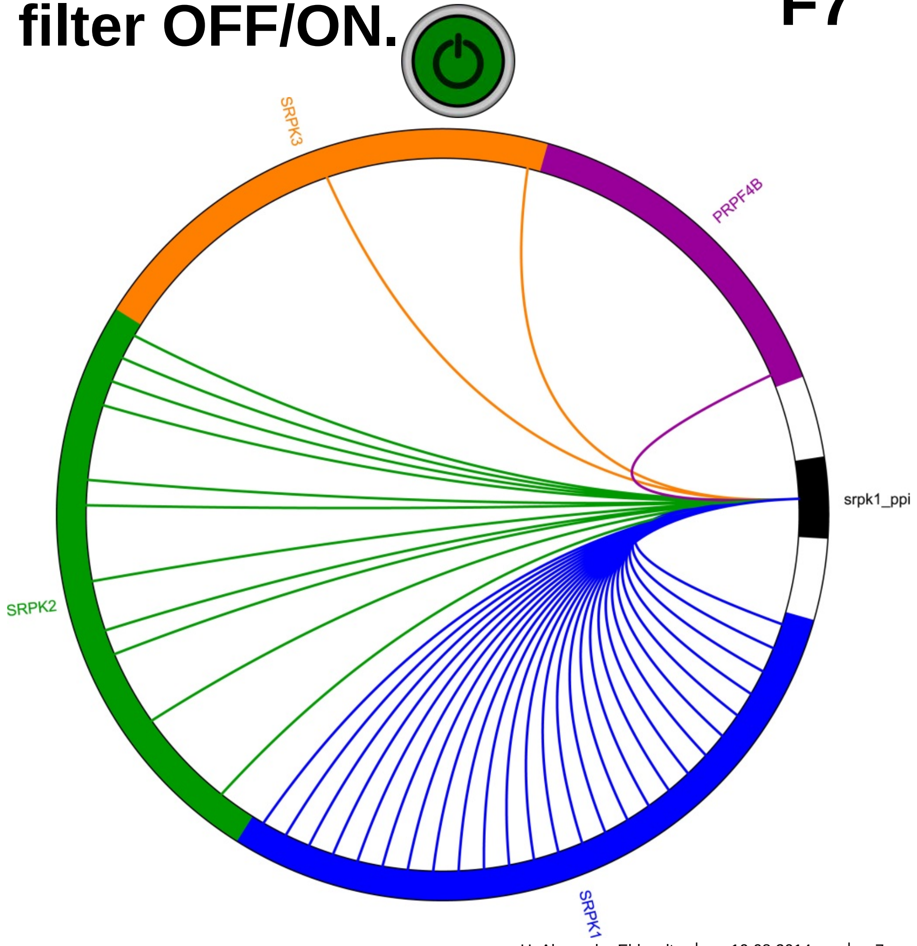

Hobbs et al. unpublished.

Kinase interactors vs. kinase interactors: Reappearance filter OFF/ON.

F8

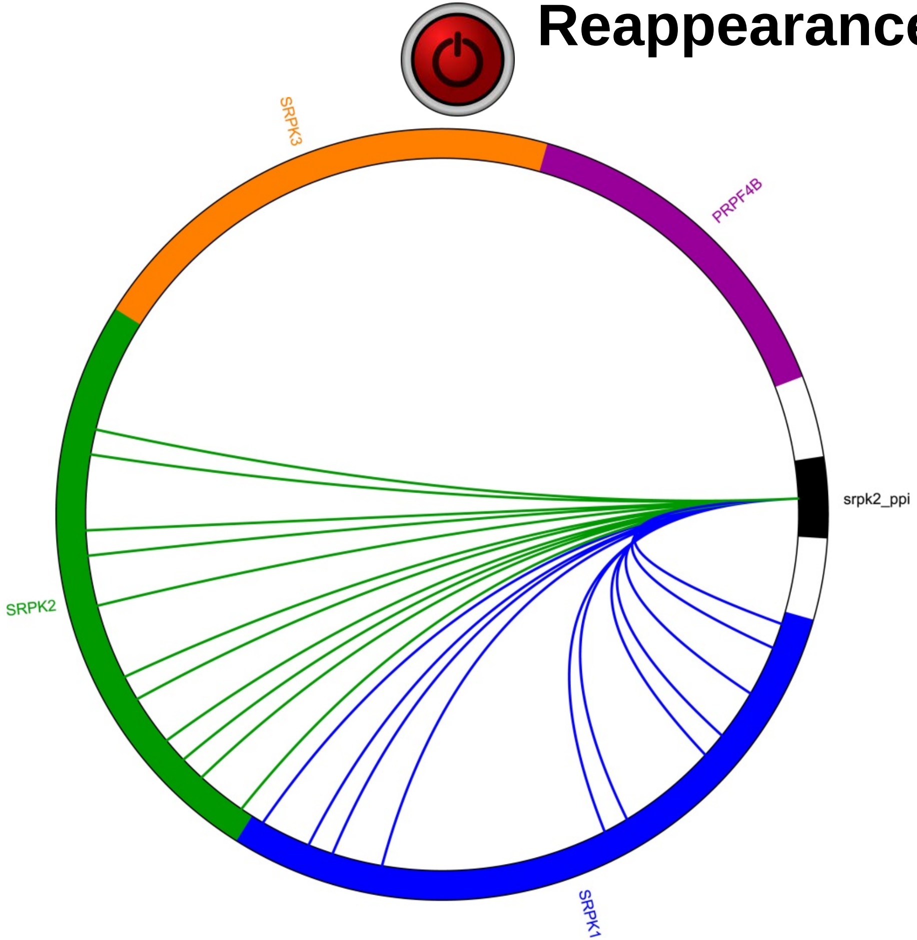

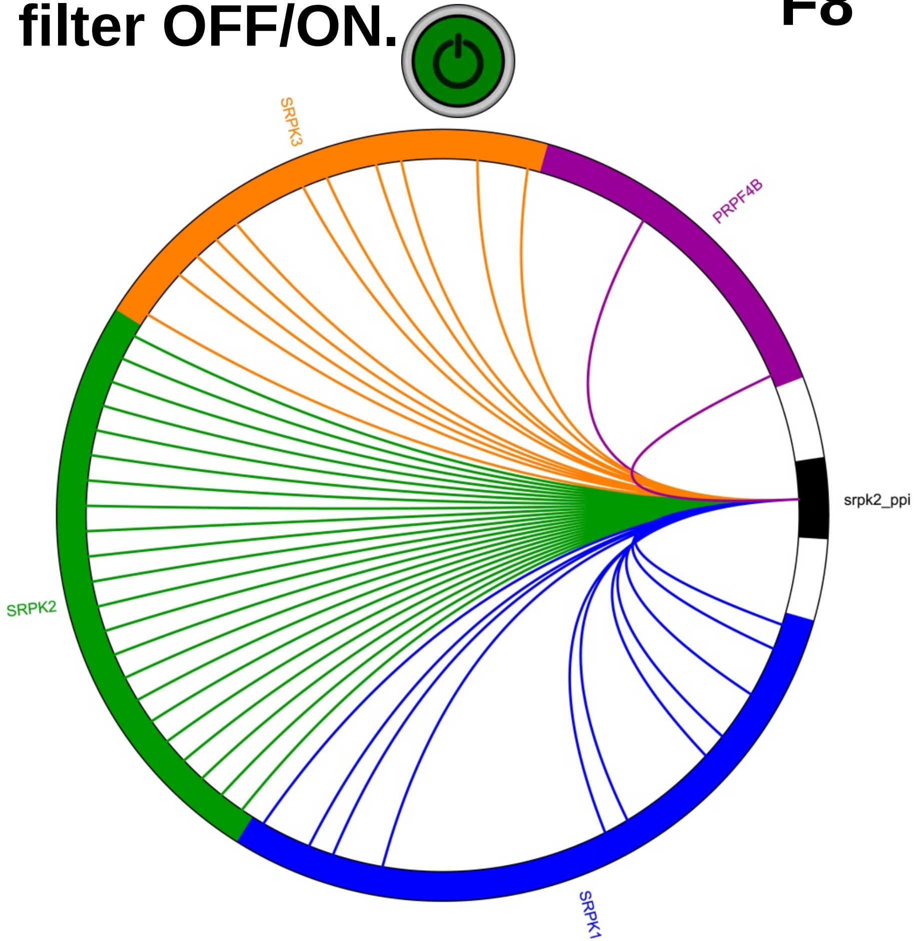

Hobbs et al. unpublished.

Kinase interactors vs. kinase interactors: Reappearance filter OFF/ON.

F9

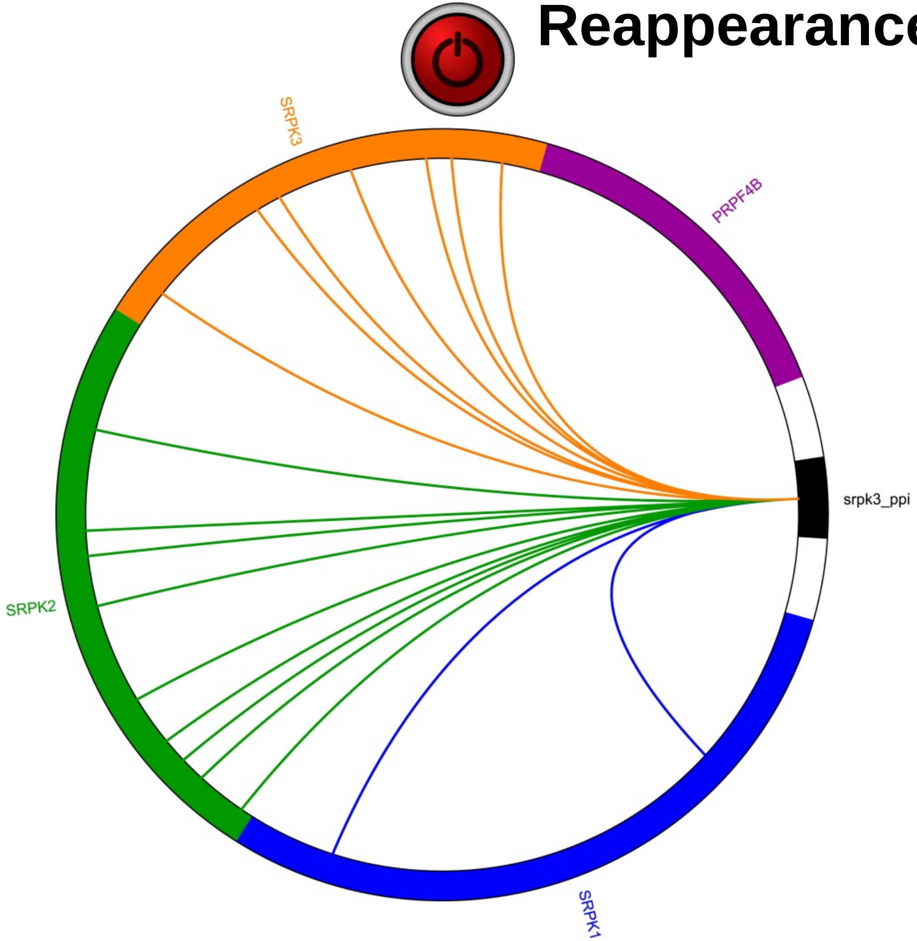

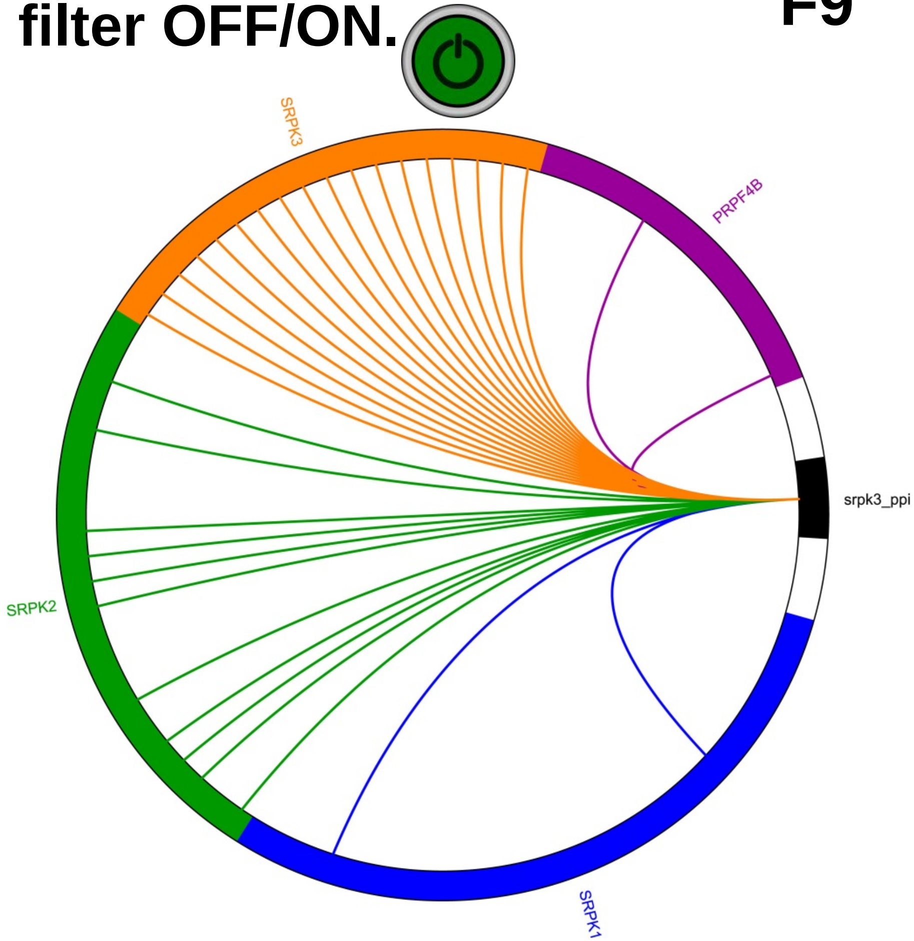

Hobbs et al. unpublished.

Kinase interactors vs. kinase interactors: Reappearance filter OFF/ON.

F10

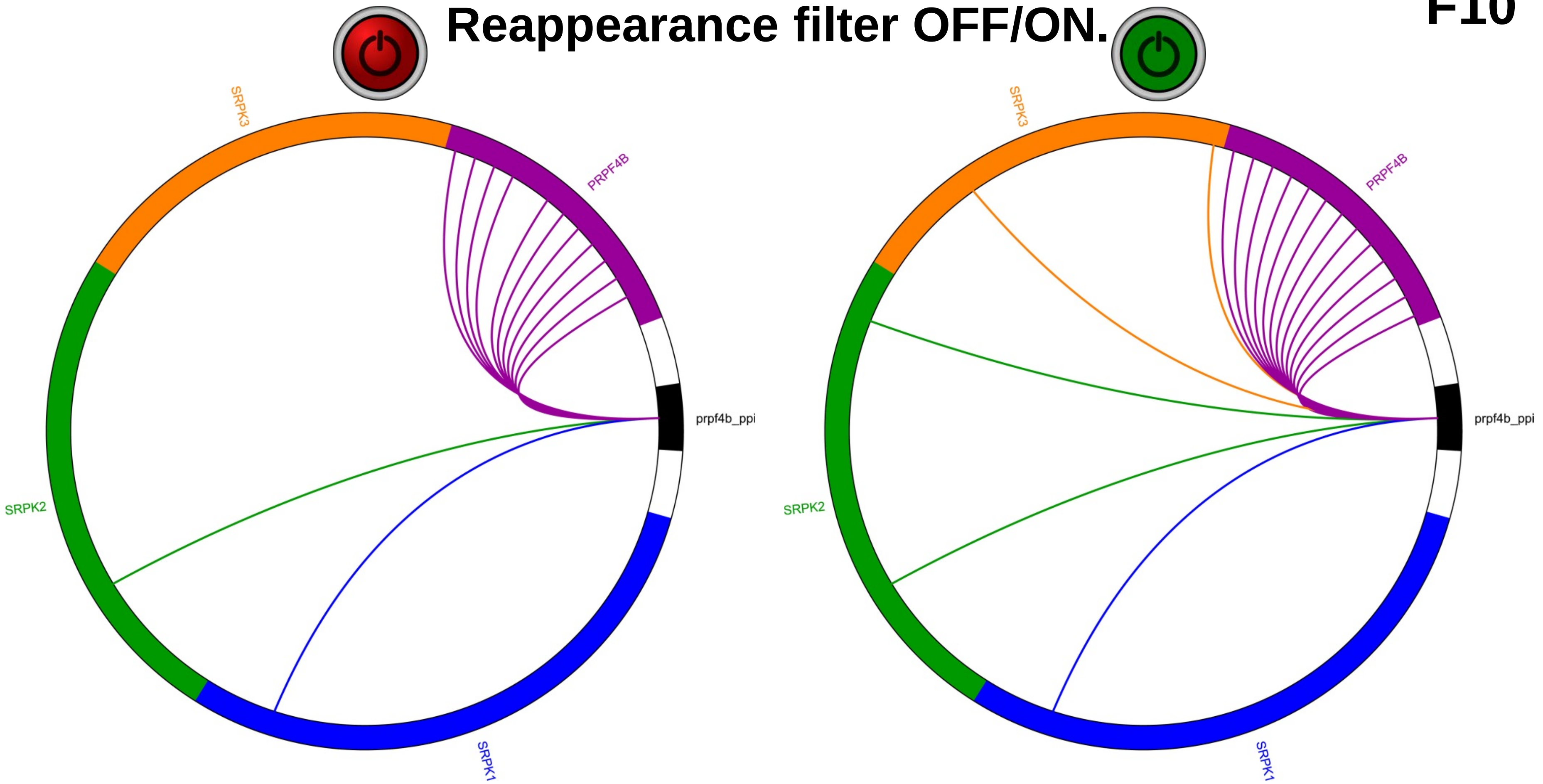

Hobbs et al. unpublished.

Integrating two proteomics datasets.

MOLECULAR AND CELLULAR BIOLOGY, July 2011, p. 2667–2682 Vol. 31, No. 13
0270-7306/11/$12.00 doi:10.1128/MCB.05266-11
Copyright © 2011, American Society for Microbiology. All Rights Reserved.

Semiquantitative Proteomic Analysis of the Human Spliceosome via a Novel Two-Dimensional Gel Electrophoresis Method[∇]§

Dmitry E. Agafonov,[1] Jochen Deckert,[1]† Elmar Wolf,[1]‡ Peter Odenwälder,[1] Sergey Bessonov,[1] Cindy L. Will,[1] Henning Urlaub,[2] and Reinhard Lührmann[1]*

Department of Cellular Biochemistry[1] and Bioanalytical Mass Spectrometry Group,[2] Max Planck Institute for Biophysical Chemistry, Am Fassberg 11, D-37077 Göttingen, Germany

Time resolved
or
Ribonucleic Protein Complex (RNP) resolved

Cell Press
Open ACCESS
Cell Reports
Resource

The Protein Interaction Landscape of the Human CMGC Kinase Group

Markku Varjosalo,[1,5] Salla Keskitalo,[1,5] Audrey Van Drogen,[1] Helka Nurkkala,[4] Anton Vichalkovski,[1] Ruedi Aebersold,[1,2,3] and Matthias Gstaiger[1,2,*]
[1]Department of Biology, Institute of Molecular Systems Biology
[2]Competence Center for Systems Physiology and Metabolic Diseases
ETH Zurich, 8093 Zurich, Switzerland
[3]Faculty of Science, University of Zurich, 8006 Zurich, Switzerland
[4]Institute of Biotechnology, University of Helsinki, 00014 UH, Finland
[5]Present address: Institute of Biotechnology, University of Helsinki, 00014 UH, Finland
*Correspondence: gstaiger@imsb.biol.ethz.ch
http://dx.doi.org/10.1016/j.celrep.2013.03.027

Hobbs et al. unpublished.

Integrating two proteomics datasets: **Time resolved.**

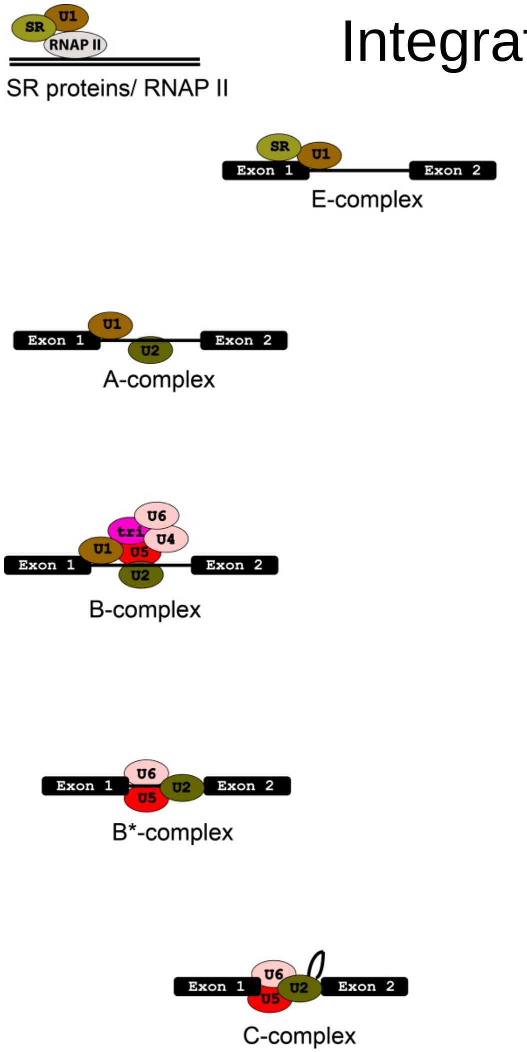

F11

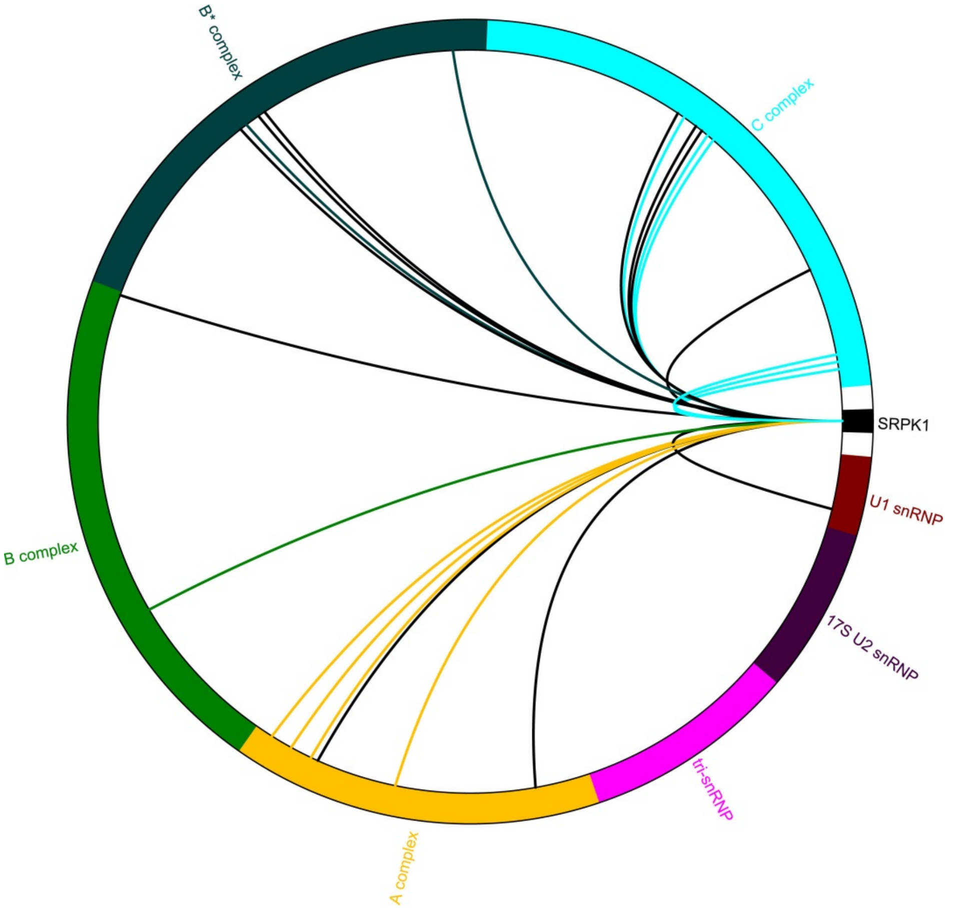

Hobbs et al. unpublished.

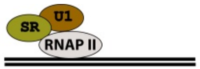

SR proteins/ RNAP II

Integrating two proteomics datasets: **Time resolved.**

F12

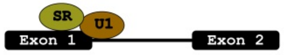

E-complex

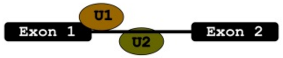

A-complex

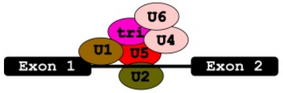

B-complex

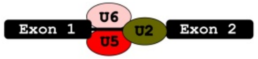

B*-complex

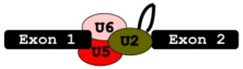

C-complex

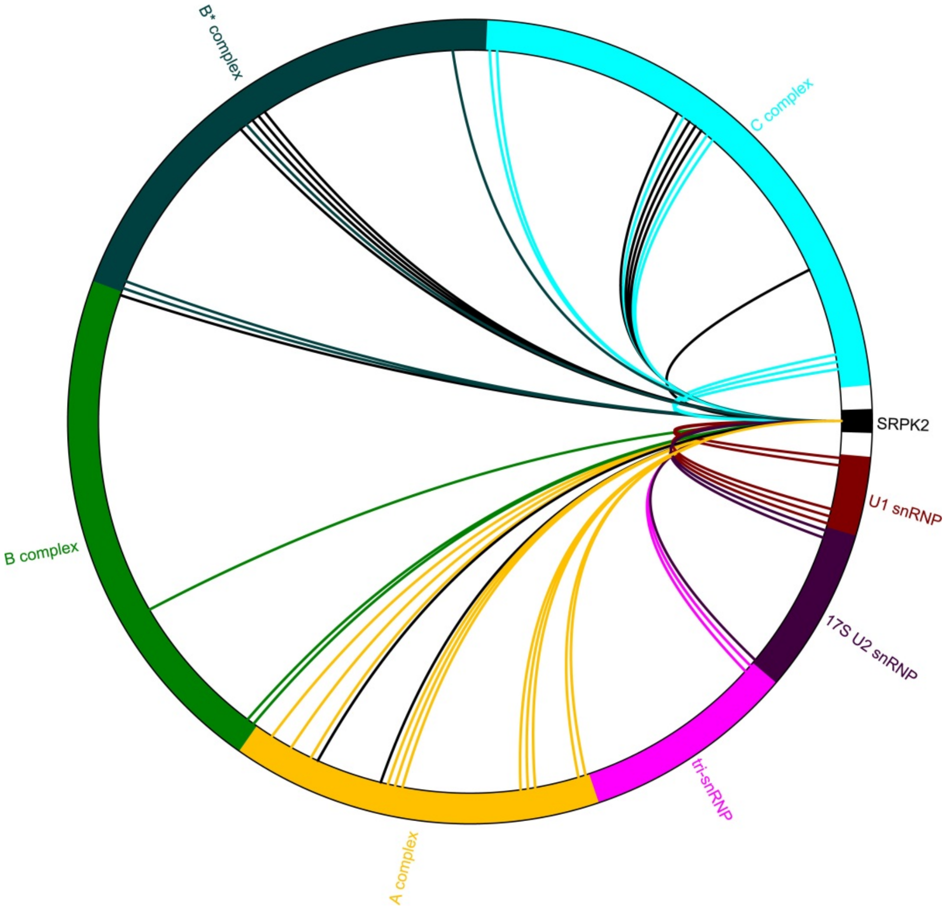

Hobbs et al. unpublished.

Integrating two proteomics datasets: **Time resolved.**

F13

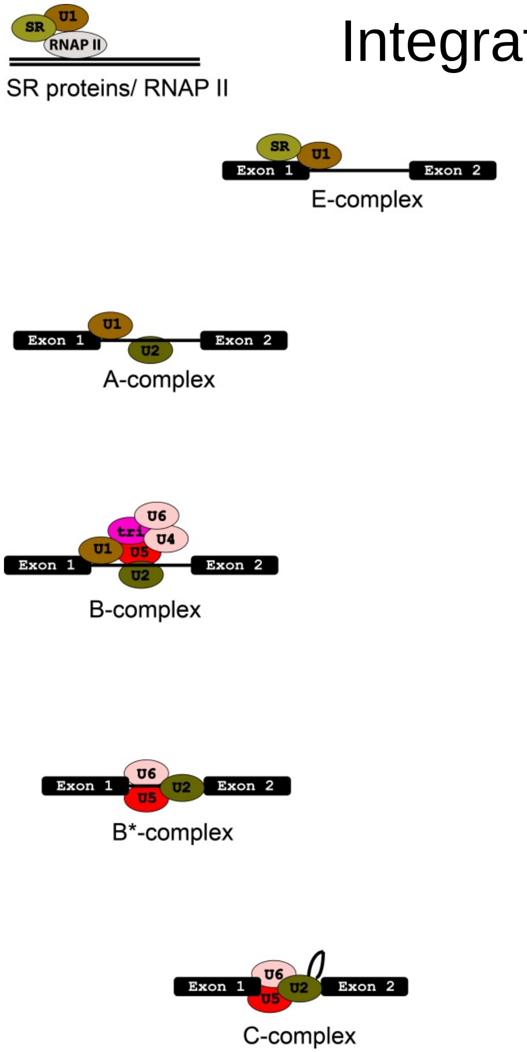

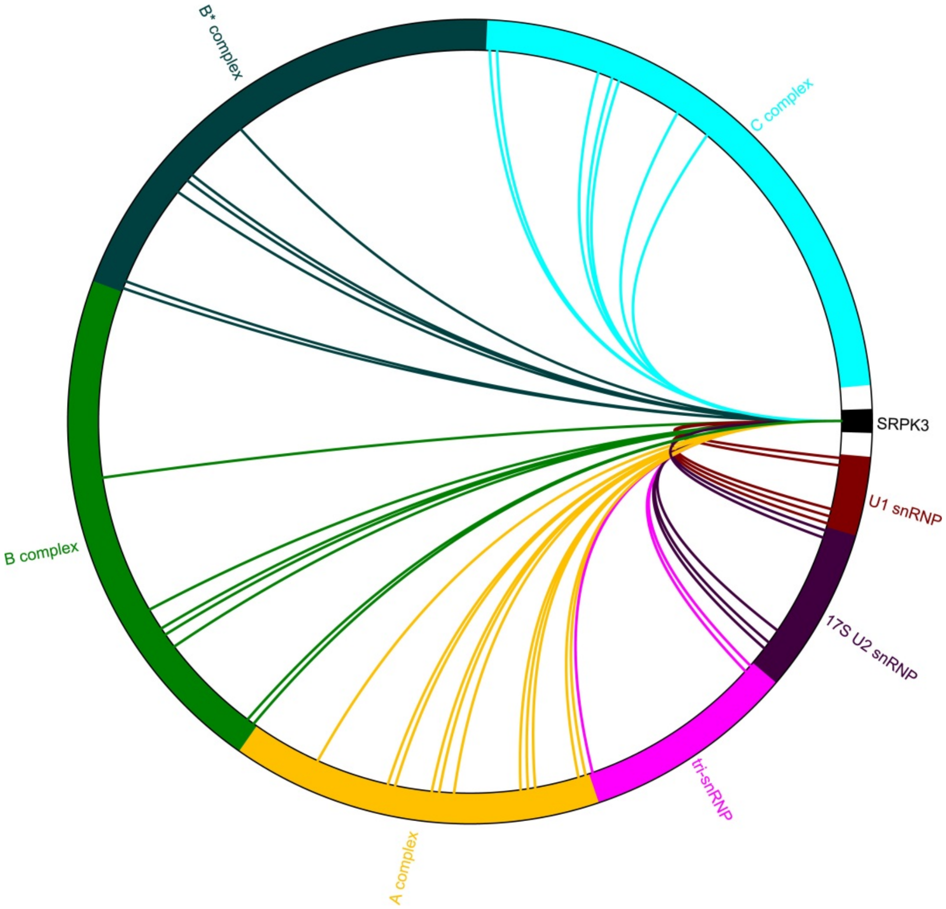

Hobbs et al. unpublished.

Integrating two proteomics datasets: **Time resolved.**

F14

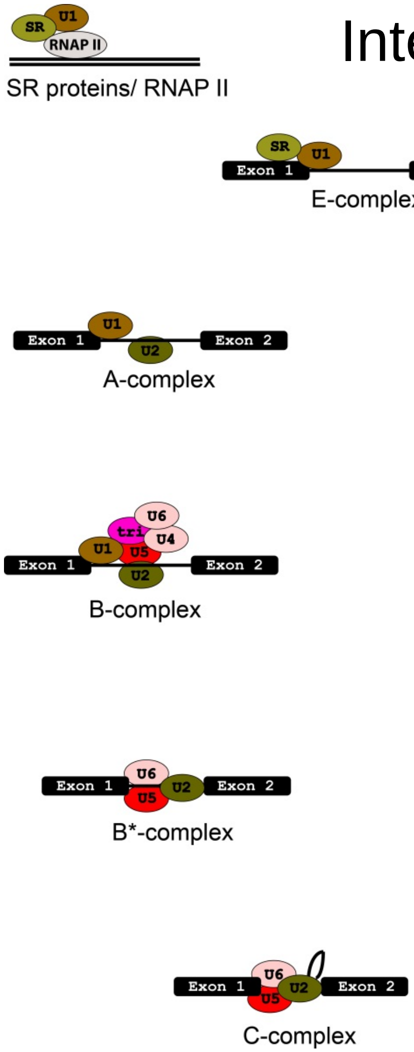

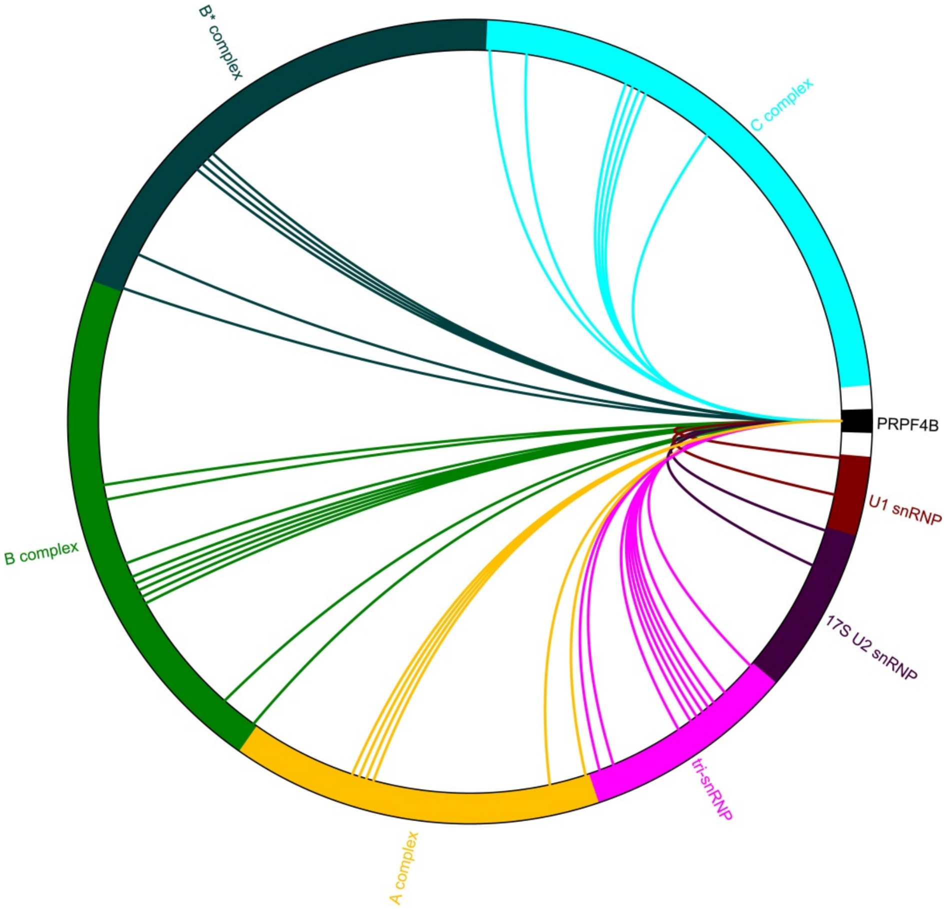

Hobbs et al. unpublished.

Integrating two proteomics datasets: **RNP resolved.**

F15

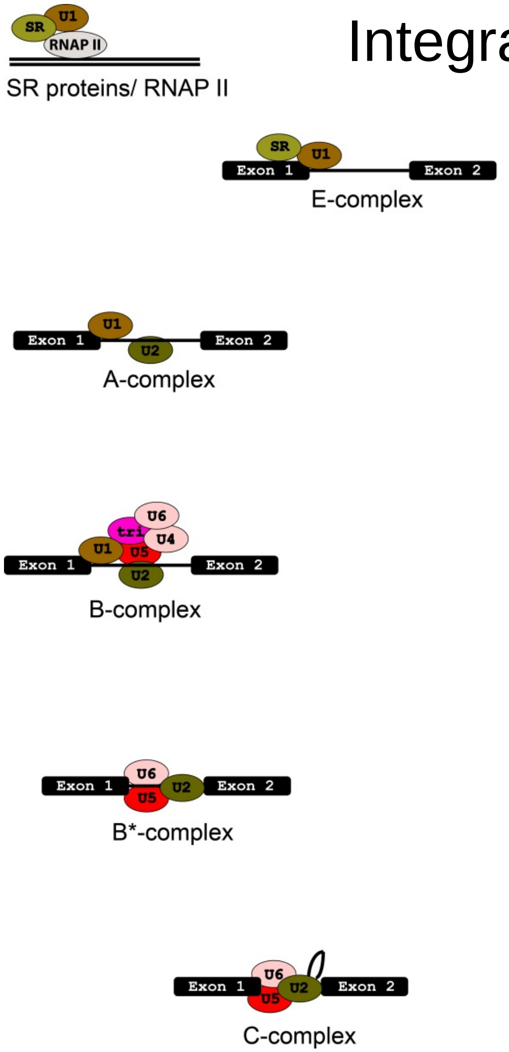

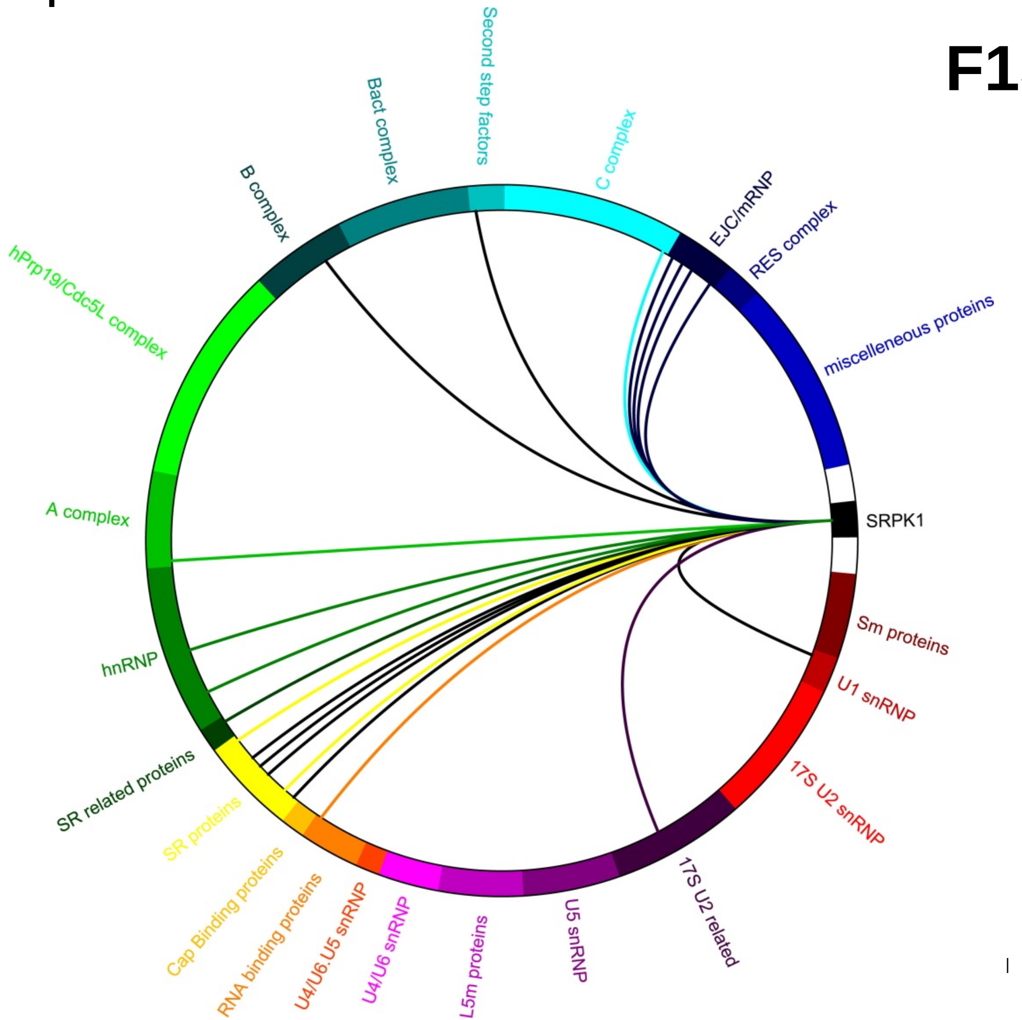

Hobbs et al. unpublished.

Integrating two proteomics datasets: **RNP resolved.**

F16

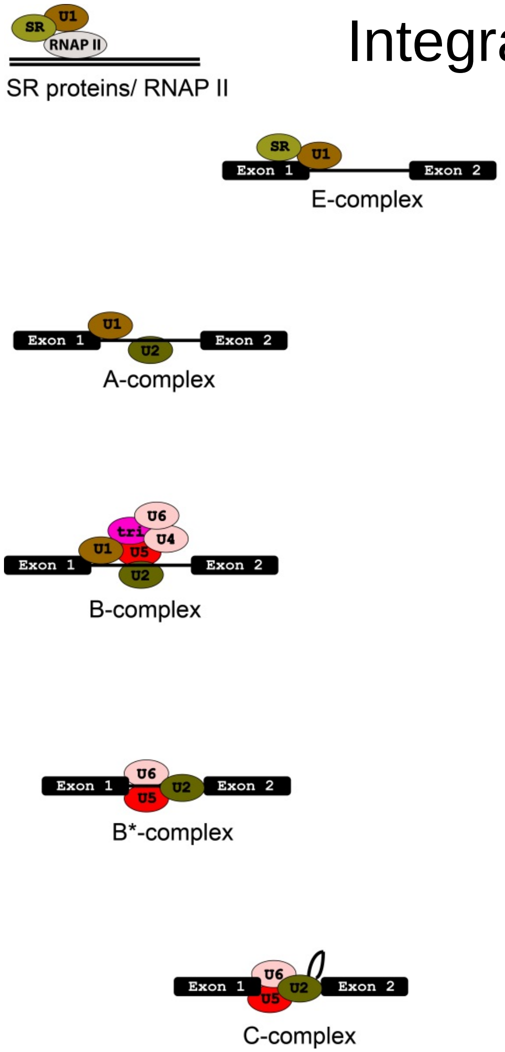

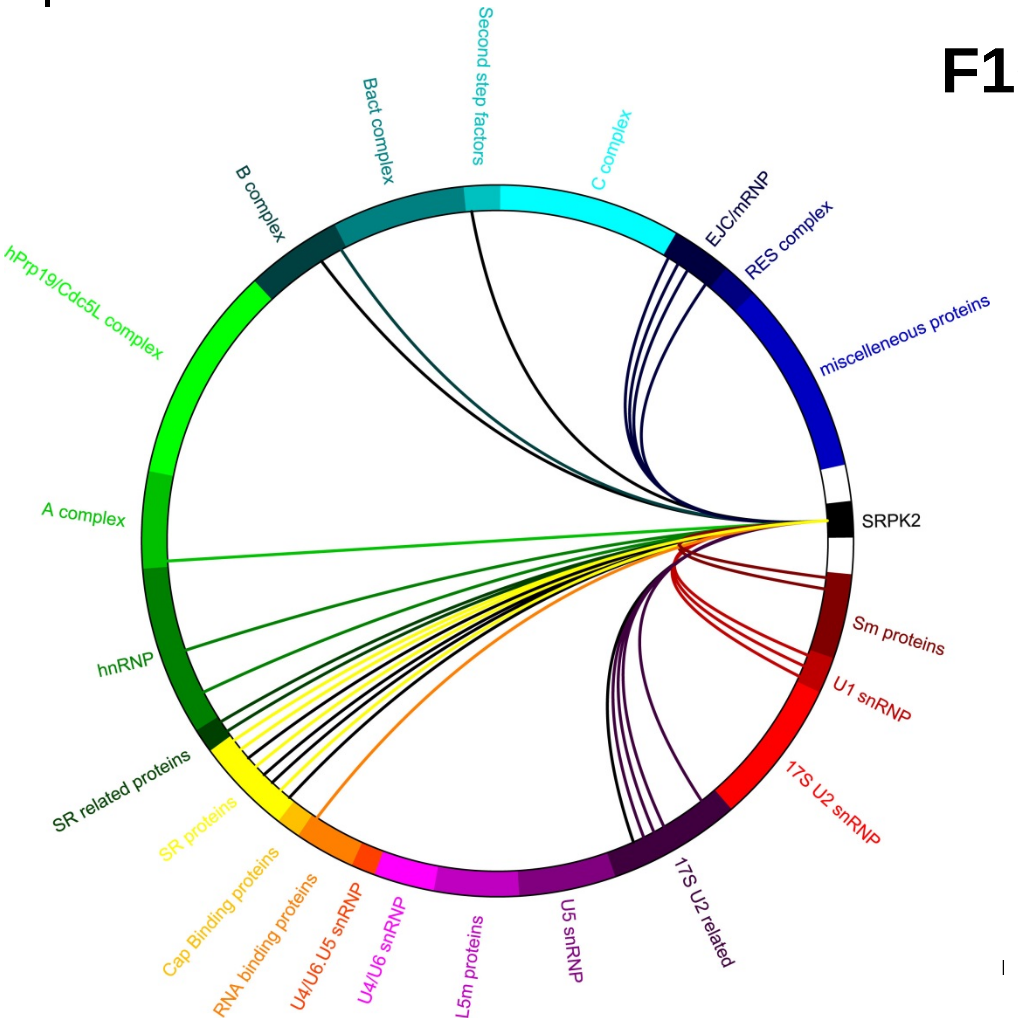

Hobbs et al. unpublished.

Integrating two proteomics datasets: **RNP resolved.**

F17

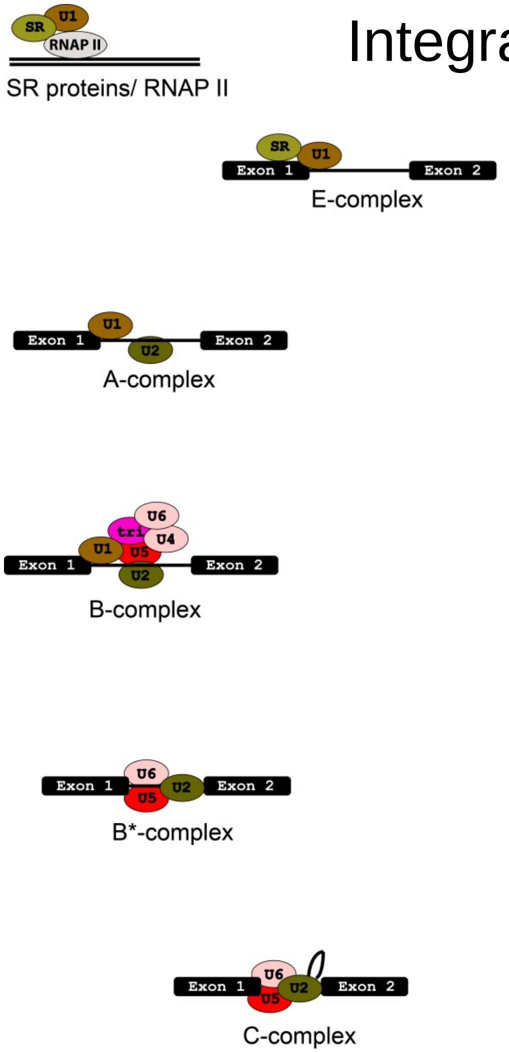

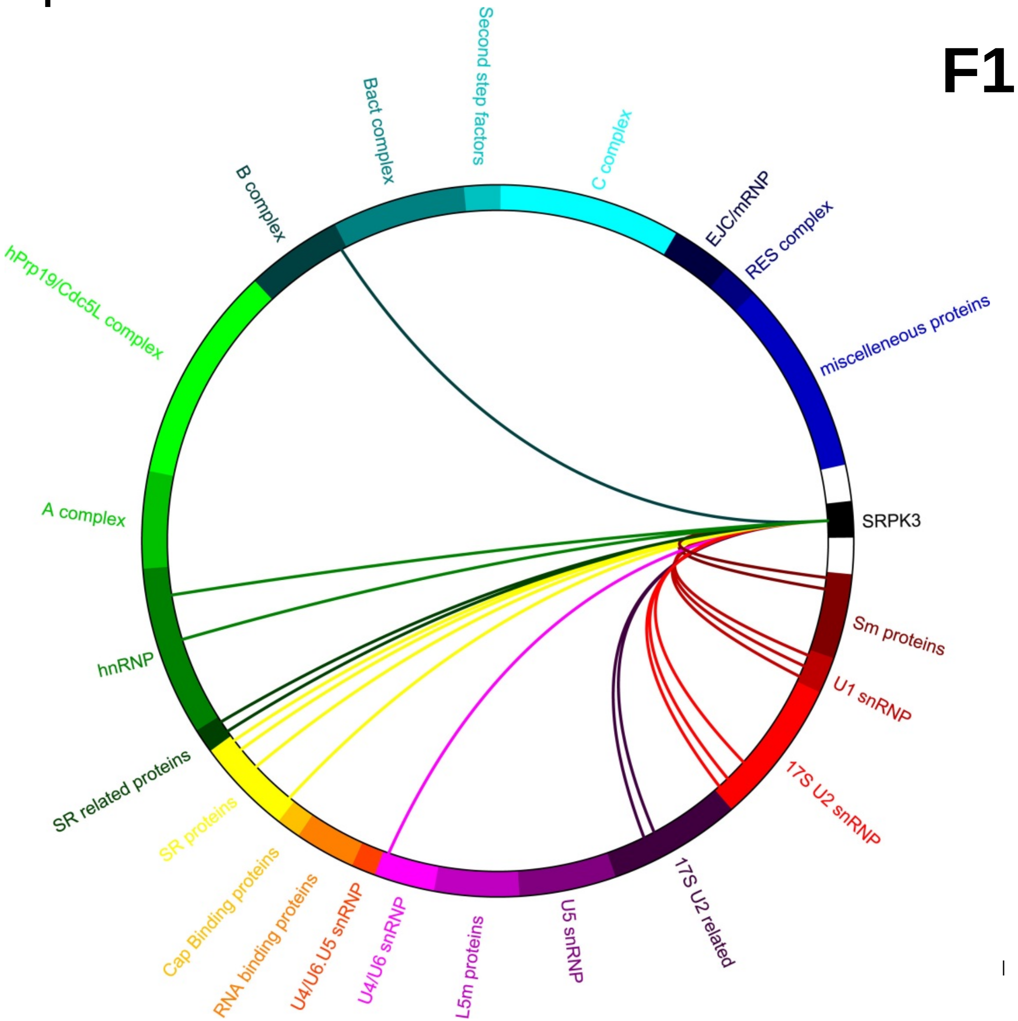

Hobbs et al. unpublished.

Integrating two proteomics datasets: **RNP resolved.**

F18

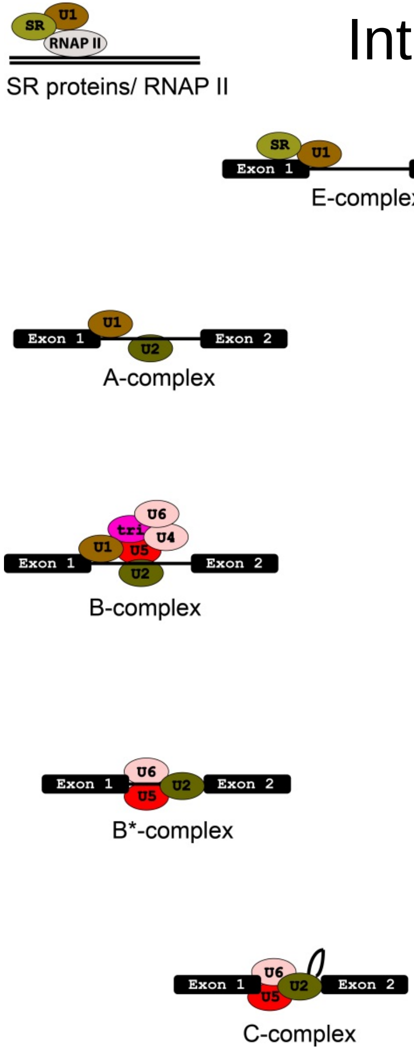

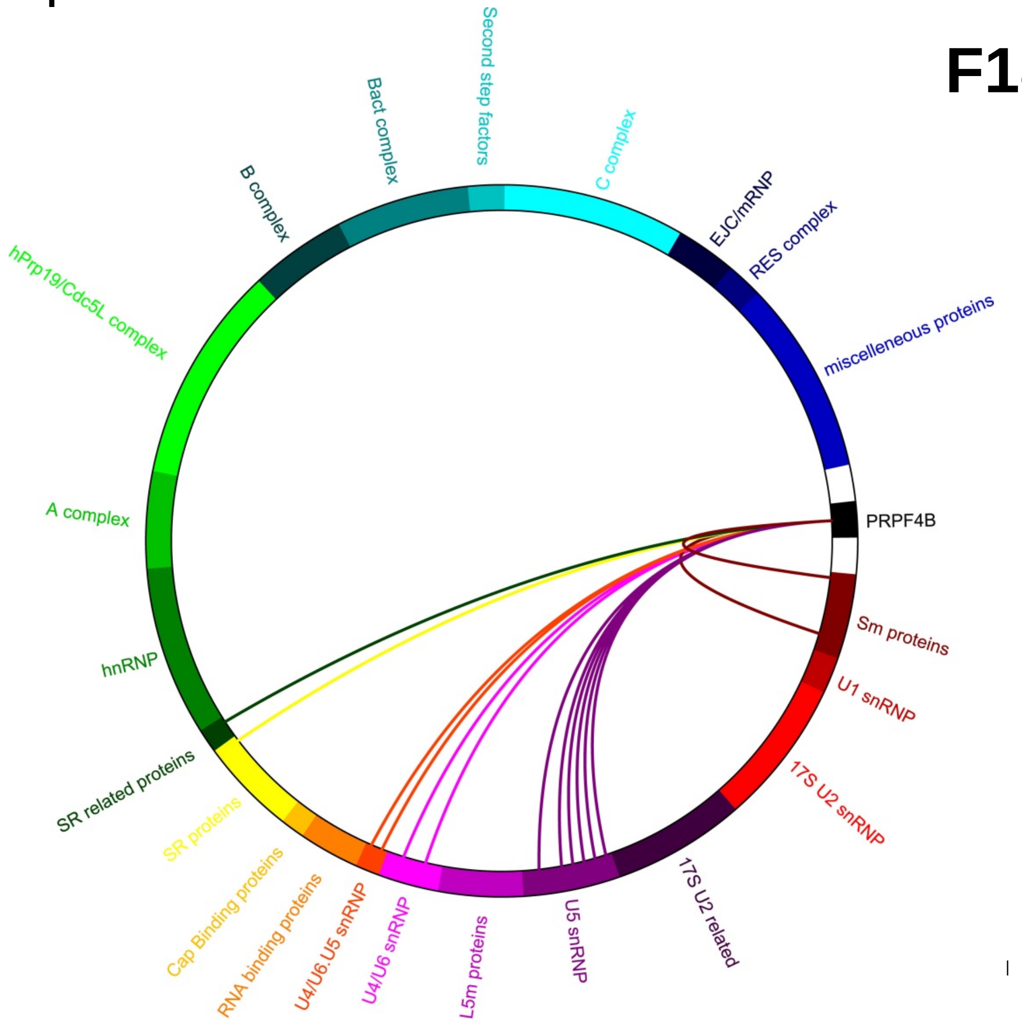

Hobbs et al. unpublished.
